# Functional Neural Architecture of Working Memory in Musicians: An ALE Meta‐Analysis and Review

**DOI:** 10.1002/wcs.70036

**Published:** 2026-06-14

**Authors:** Lee Wolff, Yixue Quan, William Forde Thompson, Oliver Baumann

**Affiliations:** ^1^ Bond University Gold Coast Queensland Australia; ^2^ Macquarie University Sydney New South Wales Australia

**Keywords:** brain mapping, cognitive reserve, functional neuroimaging, working memory

## Abstract

Musical expertise is often associated with enhanced cognitive abilities, underpinned by neural mechanisms unique to musicians. Research has provided evidence of associations between musical training and functional aspects of cognition. However, the neural changes associated with musical expertise, particularly in relation to working memory, remain poorly understood. To address this gap, we conducted an activation likelihood estimation (ALE) meta‐analysis to examine differences in functional neural activity between musicians and non‐musicians during working memory tasks. Meta‐analytic connectivity modeling (MACM) was conducted to explore broader networks of co‐activation associated with regions of interest identified during the initial ALE meta‐analysis. Nine functional magnetic resonance imaging (fMRI) studies, including 228 participants (113 musicians and 115 non‐musicians), were included in the final analysis. Musicians exhibited hyperactivations in the right medial frontal gyrus and hypoactivations across the right middle occipital gyrus, the right precentral gyrus, the left inferior parietal lobule, the right claustrum, and the left cerebellum during working memory tasks. MACM revealed distributed networks of co‐activation with links to various cognitive functions across regions of interest uncovered by the ALE analysis. Based on these findings, we propose that distinct functional networks and enhanced neural efficiency in musicians may support working memory performance across the lifespan.

This article is categorized under:

Neuroscience > Cognition
Psychology > Development and Aging
Cognitive Biology > Cognitive Development

## Graphical abstract

A Summary of ALE Meta‐Analytic Findings on the Neural Correlates Underlying Working Memory in Musicians. Figure displays a 3D rendering of regions with significantly increased (M > NM; red) and decreased (NM > M; blue) functional activation in musicians during working memory tasks, as indicated by ALE meta‐analysis.

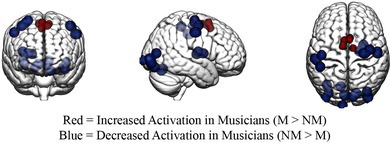

## 1 Introduction

Musical training is commonly used as a model for investigating neuroplasticity, which can be understood as the brain's ability to reorganize its structure and function in response to lifetime experiences and environmental exposures (Dinse 2020). This adaptive process can help the brain better cope with ongoing demands in the external world (Dinse 2020). Musical expertise requires advanced auditory processing to perceive melodic, harmonic, timbral, rhythmic, and emotional musical cues, as well as a complex combination of bimanual coordination, fine motor control, visuospatial perception, and sensorimotor integration (Herholz and Zatorre 2012; Olszewska et al. 2021). The acquisition of musical skills places a significant demand on brain regions involved in these processes, with musicians often exhibiting measurable neuroanatomical and functional differences across auditory, sensorimotor, interoceptive, and limbic areas (Criscuolo et al. 2022; Olszewska et al. 2021).

Musical activities also necessitate the recruitment of higher‐order cognitive abilities beyond those related to sensorimotor processes. For instance, mental abilities such as working memory, attention, inhibitory control, executive function, and long‐term memory are crucial for dynamically attending to auditory stimuli, maintaining performance, and recalling learned musical pieces (Miendlarzewska and Trost 2014; Wang 2022). Consistent with this, musicians often exhibit enhanced higher‐order cognitive processing across both musical and non‐musical contexts, as well as demonstrate functional and structural neural differences in brain regions responsible for implementing these cognitive functions (Criscuolo et al. 2022; Miendlarzewska and Trost 2014; Olszewska et al. 2021; Wang 2022). However, while extensive research supports the association between musical training and neurocognitive advantages, it remains questionable whether musical training directly causes such adaptations, or whether they reflect factors such as pre‐existing abilities or a tendency for musicians to engage in other cognitively stimulating activities (see Schellenberg and Lima 2024). Recent theories conceptualize the benefits of music engagement for cognitive function as attributable to a constellation of cognitive, behavioral, and motivational processes (e.g., sustained attention, goal‐directed practice, sensorimotor integration, and social engagement) that partially arise from contextual factors in structured musical activities including social interaction, emotional processing, and sensorimotor synchronization (Brancatisano et al. 2020; Fiveash et al. 2023; Wolff et al. 2023). Such processes place significant demand on higher‐order cognitive abilities and are intrinsic to musical training. Hence understanding neural differences that may arise in higher‐order cognitive functions could help to disentangle mechanisms underpinning enhanced performance among musicians, especially in abilities that may also be recruited to support contextual factors associated with musical activities.

Working memory, a multicomponent executive function responsible for manipulating and transferring information between short‐ and long‐term memory systems, is a key cognitive ability related to the various aspects of musical training (Chai et al. 2018; Grassi et al. 2025; Talamini et al. 2017). Although much of the existing evidence is derived from cross‐sectional research, where the causal role of musical training in modifying cognitive ability is difficult to determine, musicians are often noted to outperform non‐musicians in working memory tasks (Fennell et al. 2021; George and Coch 2011; Hansen et al. 2013; Schulze et al. 2012). However, these effects appear to differ as a function of stimulus type (i.e., musical, verbal, visuospatial: Grassi et al. 2025). For instance, while aspects of musical and verbal working memory are often evidenced as superior in musicians (Franklin et al. 2008; Lee et al. 2007; Tierney et al. 2008), evidence for advantages in visuospatial domains is less consistent and often negligible (Fennell et al. 2021; Franklin et al. 2008; Grassi et al. 2025; Talamini et al. 2017). As such, advantages in working memory function associated with musical training appear to be domain‐specific rather than generalized. Nevertheless, working memory remains central to both formal musical training and broader musical contexts, providing a useful framework for examining higher‐order cognitive processing among musicians.

Differences in working memory ability between musicians and non‐musicians are often mirrored by neural adaptations among musically trained individuals (Böttcher et al. 2022; Olszewska et al. 2021). For instance, functional imaging studies have uncovered several key neural correlates of working memory function in musicians that may help to understand performance advantages (Huang et al. 2010; Pallesen et al. 2010; Pau et al. 2013; Schulze et al. 2011, 2012; Sluming et al. 2007; Yamashita et al. 2022). However, while some studies indicate broader neural recruitment as a mechanism underpinning these effects (Huang et al. 2010; Schulze et al. 2011), with functional recruitment of brain areas not traditionally associated with working memory complementing enhanced function, others suggest that musician advantage may instead reflect increased neural efficiency within core working memory regions. From this perspective, musicians achieve similar or superior performance to non‐musicians while recruiting fewer neural resources from regions traditionally implicated in working memory function (i.e., prefrontal cortex, parietal cortex, medial temporal lobe, cerebellum and subcortical regions: Eriksson et al. 2015; Hoppe et al. 2014; Pau et al. 2013; Schmithorst and Holland 2004). Although it is possible that performance advantages among musicians could be a product of both complementary recruitment and increased neural efficiency, inconsistency among findings obscures the precise mechanisms underlying these effects. As such, exploring commonalities across neuroimaging studies is imperative for elucidating how musicians may implement enhanced working memory function.

Functional magnetic resonance imaging (fMRI) is a neuroimaging modality commonly used to explore task‐related changes in neural activation, measuring haemodynamic responses to regional oxygen demands through blood oxygen level‐dependent (BOLD) signals. When paired with a specific task, these signals can indicate greater activation (hyperactivation) or reduced activation (hypoactivation) relative to a control condition or group, allowing for inferences about neural efficiency or the recruitment of complementary neural resources during task completion. However, the extent to which activation patterns can be reliably interpreted is often limited by small sample sizes and inconsistent methodological approaches across neuroimaging studies, hindering the generalizability of this research (Müller et al. 2018). The existing inconsistency among neuroimaging literature on musical training and higher‐order cognitive functions further exacerbates these limitations and emphasizes the need for consolidating findings within this area. Coordinate‐based meta‐analysis is commonly used to address such limitations, statistically identifying convergence across fMRI studies through pooled activation data (Eickhoff et al. 2012; Müller et al. 2018). By synthesizing findings across studies, this approach enhances statistical power and reduces the likelihood of spurious results.

### 1.1 Research Aims and Hypotheses

The present study employed a coordinate‐based meta‐analysis using activation likelihood estimation (ALE) to explore similarities and differences in brain activation patterns associated with the neural implementation of working memory in musicians and non‐musicians. Further, meta‐analytic connectivity modeling (MACM) was used to explore network‐level co‐activations with regions of interest identified during the initial ALE meta‐analysis. Finally, a behavioral/paradigm analysis was conducted to explore behavioral functions associated with uncovered regions of interest. The aims of this approach were twofold. First, to determine whether musicians engage complementary brain pathways outside of core working memory regions, including the prefrontal cortex, parietal cortex, medial temporal lobe, basal ganglia, and cerebellum (Eriksson et al. 2015), to support working memory function. Second, to assess whether musicians exhibit reduced reliance on these core regions while achieving equal or superior cognitive performance compared to non‐musicians. Hence, we hypothesized that musicians would exhibit hyperactivations in brain regions not typically associated with working memory, indicating broader neural recruitment to support task implementation. We also hypothesized that musicians would exhibit hypoactivation in areas generally associated with working memory function when performing equal or better than non‐musicians on behavioral tasks, suggesting increased neural efficiency among musicians.

## 2 Methods

### 2.1 Search Strategy and Eligibility Criteria

**FIGURE 1:**
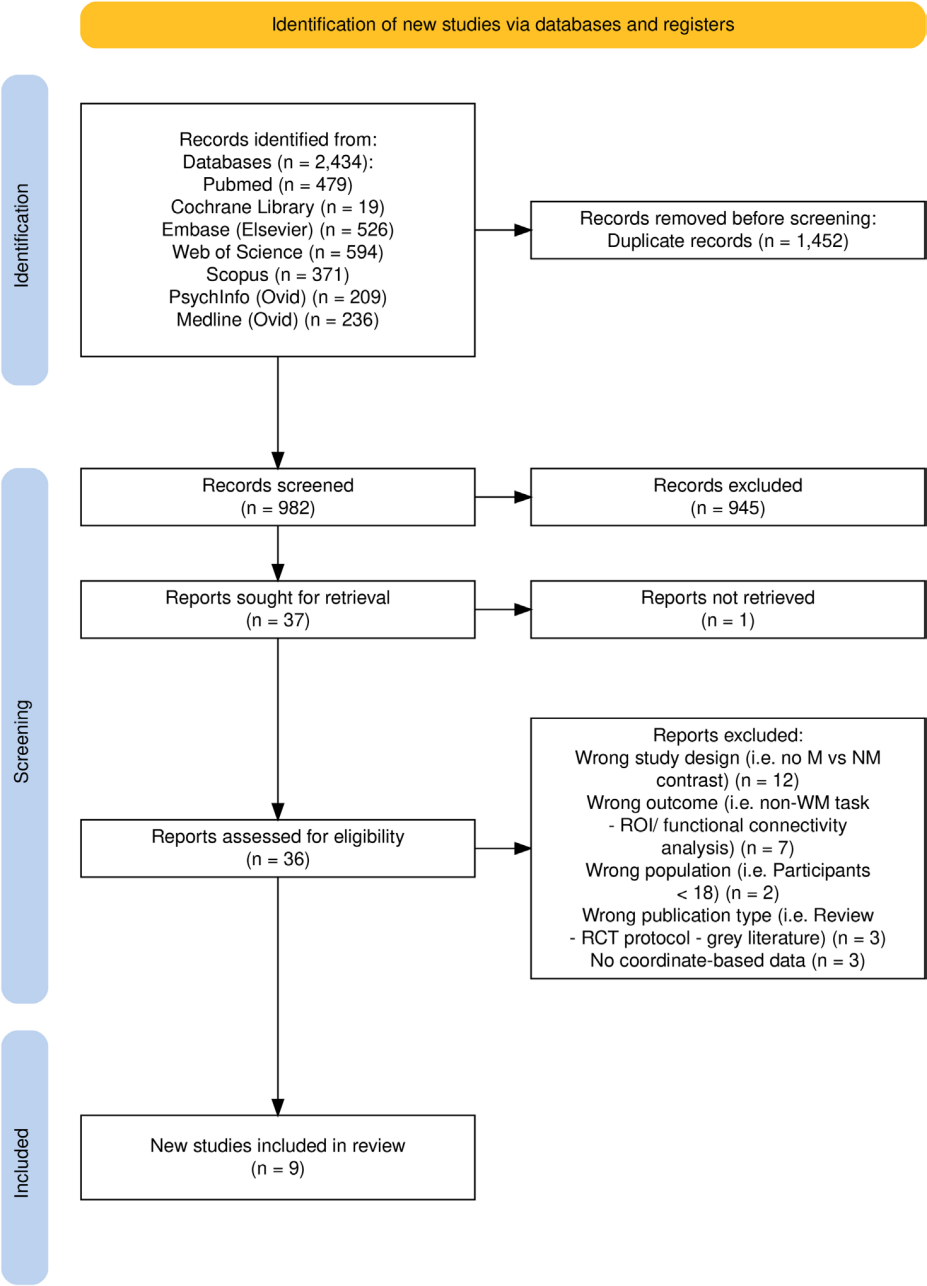
PRISMA flowchart of article search and selection procedures.

The present study was conducted in accordance with the Preferred Reporting Items for Systematic Reviews and Meta‐Analysis (PRISMA) guidelines (Page et al. 2021). “The Evidence Review Accelerator (TERA)” an automated systematic review tool (Clark, Glaziou, et al. 2020 and Clark, Sanders, et al. 2020), was employed to ensure the completeness of our approach. The literature search was conducted on 21/01/2026. Scientific databases including PubMed, Cochrane Library, Embase (Elsevier), Web of Science, Scopus, PsycInfo (Ovid), and MEDLINE (Ovid) were utilized to conduct a search of the literature with the following search terms: ((Musician* OR “Musical Expert*” OR “Music Expert*” OR “Musical Train*” OR “Music Train*” OR “Musically Trained” OR Pianist* OR Violinist* OR Guitarist* OR Drummer* OR Vocalist*) AND (Imaging OR Neuroimaging OR “Functional neuroimaging” OR fMRI OR “Functional Magnetic Resonance Imaging” OR PET OR “Positron Emission Tomography”) AND (“Working Memory” OR Memory OR Cognit* OR “Executive function*”)) . A Polyglot Search Translator Tool (Clark, Glaziou, et al. 2020 and Clark, Sanders, et al. 2020) embedded in TERA was utilized to formulate search terms for each database. The publication year was not restricted and included articles available from database inception up until the January 21, 2026. Table S1 provides further details about search terms and database results. Table S2 provides further detail on the PICO (Population/Intervention/Comparison/Outcome), and inclusion/exclusion criteria. Figure 1 depicts the study selection process delineated through a PRISMA flow diagram (Haddaway et al. 2022).

A total of 2494 relevant articles were identified and extracted from the 7 databases. An “Automated Systematic Search Deduplicator” (AsySD) was utilized to identify and remove duplicates (Hair et al. 2023). The remaining 1045 unique citations were imported into Rayyan (Ouzzani et al. 2016), where the screening tool detected and removed a further 63 duplicate articles. Titles and abstracts of the remaining 982 articles were assessed against selection criteria by two independent reviewers (L.W. and Y.Q.). Studies were considered for full‐text screening if they (1) cross‐sectionally compared functional brain activity between musicians and non‐musicians on in‐scanner working memory tasks, (2) were conducted in healthy adult populations, (3) were published in English, (4) utilized task‐based or positron emission tomography (PET). Studies were excluded if they (1) were review articles, book chapters or meta‐analyses that did not include original experimental data, (2) focused on structural neuroimaging (i.e., MRI, DTI), (3) utilized neuroimaging modalities that are incompatible with task‐based fMRI or PET (i.e., EEG, MEG, SPECT, fNIRS, resting‐state fMRI), (4) focused on pediatric (< 18) or clinical populations (i.e., neurological or psychiatric populations), (5) contrasted special groups of musicians (i.e., Absolute Pitch vs. Relative Pitch Musicians), (6) employed functional connectivity analysis, or (7) utilized region‐of‐interest (ROI) analyses.

A total of 26 conflicts (2.6%) were identified after the initial screening phase. These conflicts were discussed and resolved through consensus. In cases where consensus could not be reached, a third reviewer (O.B.) was involved to ensure the decision was impartial and aligned with the eligibility criteria. Following this, 37 articles were sought for retrieval. One article was unavailable for retrieval as the journal archived publications prior to 2014. Authors were contacted but were unable to provide the article. The remaining 36 articles underwent a full‐text review against eligibility criteria by two reviewers (L.W. and Y.Q.). In this phase, studies considered for final inclusion were required to report functional neuroimaging data as stereotactic coordinates using the Talairach or Montreal Neurological Institute (MNI) three‐dimensional coordinate system. Authors were contacted when data were not reported in this format. Of the two authors contacted, neither was able to provide the requested data. A total of nine eligible articles were included in the final meta‐analysis. Table S3 details the reasons for exclusion of each article assessed during the full‐text review.

### 2.2 Data Extraction

Data were extracted for the following variables in the included studies: (1) first author, (2) year of publication, (3) sample demographics (i.e., number of participants [both musicians and non‐musicians], age, and gender ratio), (4) years of musical training, (5) age of musical training onset, (6) musical instrument, (7) details about in‐scanner neuroimaging task, (8) details about MRI‐system (field strength, model, head‐coil, image acquisition parameters, repetition time [TR], echo time [TE], voxel size, analysis method, and software). The primary outcome data during extraction were stereotactic coordinates comparing neural activation between musicians and non‐musicians during working memory tasks, reported in the Talairach or Montreal Neurological Institute (MNI) three‐dimensional coordinate system. These data were utilized for the ALE meta‐analysis.

### 2.3 ALE Analysis

Activation Likelihood Estimation analysis was implemented through GingerALE version 3.0.2, a widely used tool for coordinate‐based meta‐analyses. ALE techniques statistically assess the convergence of activation foci extracted from functional neuroimaging studies. Foci coordinates (*x*, *y*, *z*) from each study are used to centre spatial probabilities (Eickhoff et al. 2012, 2016). The ALE algorithm then examines to what extent activation foci converge across independent fMRI studies by computing the union of activation probabilities for each voxel (Eickhoff et al. 2012). These foci are then tested against the null distribution of random spatial association between studies to differentiate true foci convergence from noise created by random foci clustering (Eickhoff et al. 2016). GingerALE mitigates risk of bias by employing an uncertainty of random effects model. GingerALE also models Gaussian probability distributions with full‐width half‐maximum (FWHM) calculations to allow for spatial smoothing across nearby voxels, reducing the impact of random variability within‐ and between‐subjects. The width of resulting Gaussian distributions reflects the size of the imputed study, where larger samples are weighted more heavily than smaller samples (Eickhoff et al. 2009).

Our analysis employed a cluster‐level family wise error (FWE) threshold to account for false positive results that may arise from multiple comparisons within the same voxel. Statistical significance of ALE outcomes was determined using a cluster‐forming threshold of *p* = 0.05 and a cluster level FWE correction of *p* < 0.05 based on 1000 permutations (Eickhoff et al. 2012). These parameters represent an optimal balance between sensitivity and specificity in smaller meta‐analyses (*n* = 9), mitigating the risk of non‐significant convergence (Eickhoff et al. 2016). When necessary, a Lancaster transform (icbm2tal) executed through GingerALE was utilized to convert Talairach coordinates to MNI space, standardizing data within the same stereotaxic space. For studies with multiple experiments or contrasts in one study group, or separate articles that utilized the same sample, coordinates from relevant contrasts were pooled, adhering to prior validated approaches (Müller et al. 2018; Turkeltaub et al. 2012). Computed results were labeled using the standard MNI atlas implemented within GingerALE. MRIcroGL v1.2 was utilized to visualize ALE activations via the MNI atlas (https://www.nitrc.org/projects/mricrogl/).

### 2.4 Statistical Contrasts

The current study applied the ALE meta‐analytic technique to two between‐group contrasts to assess differences in neural activity between musicians (M) and non‐musicians (NM) on working memory tasks. These contrasts were employed to assess directionality of M vs. NM activations. Contrast one pooled foci reporting higher brain activity in musicians during working memory tasks (M > NM), whereas contrast two pooled foci reporting lower brain activity in musicians during working memory tasks (in other words, greater brain activity in non‐musicians: NM > M).

### 2.5 Meta‐Analytic Connectivity Modeling

To complement our approach, MACM was employed using Sleuth v3.0.4 (Laird et al. 2005). MACM is a technique that draws upon a large database of published brain activation data to identify patterns of co‐activation relative to functional regions of interest (ROIs) (Laird et al. 2013; Robinson et al. 2010). In our approach, ROIs derived from the initial ALE meta‐analyses were utilized as “seed regions.” Sleuth employs these seeds to search the BrainMap database for studies in which activation occurred within each seed region, regardless of the task administered during scanning. Coordinate‐based data are then extracted from each relevant study, including activation foci outside the seed region. Convergences in regions co‐activated with the seed are then analyzed using ALE. Seed regions are expected to show high convergence across studies, while surviving convergent activations outside the seed indicate a pattern of co‐activation observed in the literature. This provides a data‐driven characterization of “connectivity” between the ROI and whole‐brain networks.

Mango v4.1 was utilized to create spherical ROIs with a 6 mm radius, centred on peak ALE coordinates (http://rii.uthscsa.edu/mango//userguide.html). The selection of a 6 mm radius for functional ROIs was chosen to maintain consistency with existing MACM pipelines (Kotkowski et al. 2018). These ROIs were entered into Sleuth to search the BrainMap platform for co‐activation data. Data were only considered for the MACM if they were extracted from adult (> 18), non‐clinical groups during normal‐mapping experiments. Parameters in sleuth were also set to include activations only. Furthermore, to maintain consistency with the ALE analysis, only fMRI studies were included, while other imaging modalities (i.e., EEG, PET, fNIRS, etc.) were excluded. Once data were extracted, GingerALE 3.0.2 was utilized to conduct coactivation analyses using the ALE algorithm. Statistical significance of coactivated regions was determined using a cluster‐forming threshold of *p* = 0.05, a cluster level FWE correction of *p* < 0.001, based on 1000 permutations (Eickhoff et al. 2012). These parameters were modified from the initial ALE to increase the specificity of this analysis. Computed results were labeled using the standard MNI atlas implemented within GingerALE. MRIcroGL v1.2 was utilized to visualize MACM activations via the MNI atlas (https://www.nitrc.org/projects/mricrogl/).

### 2.6 Behavioral/Paradigm Analysis

We utilized Mango Behavioral Analysis v4.1 and Paradigm Analysis v2.0 plugins to functionally decode and profile the significant clusters from the ALE analyses (Lancaster et al. 2012). Both plugins draw from metadata stored on the BrainMap platform, which organize functional imaging activation data according to behavioral domains and paradigm classes. Hence, ROIs uncovered by ALE can be mapped to cognitive functions associated with given behavioral domains and paradigm classes characterized by the BrainMap platform (Fox et al. 2005; Fox and Lancaster 2002; Laird et al. 2005; Vanasse et al. 2018). There are five behavioral domains on the platform, including Action, Cognition, Emotion, Interoception and Perception. Each domain also contains a range of subdomains, with 59 subdomains currently listed on the platform (e.g., Cognition: Memory, Cognition: Language). Meanwhile, 101 common paradigm classes are used to characterize experimental imaging paradigms of studies listed on the platform (i.e., Stroop, Go/No‐Go). To quantitatively assess the association between an ROI and a given domain/paradigm, the plugins compare the likelihood of activation in a cluster for that domain/paradigm against the clusters baseline activation probability. *Z*‐scores are computed for each behavioral subdomain or paradigm class and assessed for significance with a binomial test, with *z*‐scores ≥ 3.0 being considered significant (*p* < 0.05, Bonferroni corrected for multiple comparisons: Lancaster et al. 2012). The result of this is a statistically tested, data‐driven behavioral profile of ROIs drawn from ALE analysis.

### 2.7 Evaluation of Robustness

Although ALE meta‐analyses aim to overcome limitations present in individual imaging studies, they remain subject to biases common to meta‐analytic approaches. Publication bias is a key concern, as studies reporting significant findings are more likely to be published than those with non‐significant results, potentially distorting meta‐analytic outcomes (Acar et al. 2018). To assess and mitigate risk of bias in the current study, three precautions were implemented. First, we examined the correlation between sample size and the number of reported foci in a given sample. As smaller samples are more likely to be published when results align with a priori hypotheses, a significant negative correlation may indicate the presence of publication bias (David et al. 2013). As such, we assessed both M > NM and NM > M analyses using Spearman's rho to account for sample size and deviation from normality. Both the M > NM (*ρ* = 0.05, *p* = 0.910) and NM > M (*ρ* = −0.40, *p* = 0.750) analyses indicated no significant negative correlation between sample size and number of foci. Second, a table of study‐level foci contribution was collated, identifying which experiments contributed foci to each cluster detected in the ALE analysis (see Table 2). This diagnostic characterized the sources of data underlying each activation cluster and allowed for an assessment of whether clusters were disproportionately driven by a small number of studies.

Thirdly, a Fail‐Safe N analysis (FSN: Acar et al. 2018) was conducted to assess the influence of unpublished studies on the “file drawer effect,” whereby positive results are overrepresented in published literature. The FSN approach in ALE meta‐analysis estimates the amount of contra‐evidence that can be introduced before identified activation clusters lose statistical significance. As ALE requires input data in the form of activation peaks, this is achieved by generating null or noise experiments matched to the included studies in terms of sample size and number of foci, but with foci randomly distributed across the brain (Acar et al. 2018). To generate null experiments, R code from Acar et al. (2018) was implemented in R version 4.5.1. Cluster robustness was assessed using predefined lower and upper thresholds for the number of additional studies required to alter statistical significance. The lower bound threshold directly addresses the “file drawer” problem by estimating the minimum number of unpublished null studies that a cluster must withstand before losing significance. Given estimates that up to 30% of neuroimaging studies remain unpublished (Samartsidis et al. 2019), the lower bound threshold was set to 3 for the M > NM analysis and 1 for the NM > M analysis. Meanwhile, the upper bound threshold guards against results being disproportionately influenced by a small number of contributing studies. This threshold was defined according to prior recommendations (Lo Presti et al. 2025): (Number of studies contributing to a cluster/0.1) − Total Number of studies included in ALE meta‐analysis. The results of the FSN analysis are summarized in Table 2.

## 3 Results

### 3.1 Characteristics of Included Studies

Nine articles met the inclusion criteria for quantitative ALE meta‐analysis. The included articles contained 11 experiments and a total of 228 participants. Of the participants, 113 were musicians, whereas 115 were non‐musicians. All musicians were considered experts according to included articles and were either students or graduates of a conservatory/music academy (*n* = 34), professional musicians (*n* = 11), musically trained participants of a high‐level choral group (*n* = 7) or had commenced and maintained musical practice on a regular basis since childhood (*n* = 61). All non‐musicians had little (< 3 years) or no formal musical training. Male participants constituted 49% of the sample (*n* = 111), whereas female participants constituted 51% of the sample (*n* = 117). Years of music education and age of musical onset were reported in 4 studies (44%). Musical instruments were reported across most studies (78%); one study focused solely on pianists (11%), while another was solely focused on vocalists (11%). All other studies consisted of mixed samples. Finally, all studies utilized tasks that assessed working memory. Common paradigms included verbal/tonal/visuospatial encoding and retrieval (*n* = 4), *n*‐back (*n* = 2), 3D mental rotation (*n* = 1), mental arithmetic (*n* = 1) and visuotonal transfer (*n* = 1). Table S4 details the characteristics of the included studies.

### 3.2 MRI Characteristics

All studies reported MRI system, field strength, T2 sequence, T2 repetition time, T2 echo time and analysis software. MRI scanners were either Siemens (78%), Bruker (11%), or General Electric (11%) and were either 1.5T (44%) or 3T (56%) in field strength. The most common software for MRI analysis was SPM (44%), followed by LIPSIA (22%), FSL (11%), IDL (11%) and ADNI (11%). Most studies reported coordinates in MNI space (56%), whereas the remainder reported findings in Talairach Space (44%). Further information regarding MRI scanner and analysis characteristics can be found in Table S5.

### 3.3 ALE Meta‐Analysis

**FIGURE 2:**
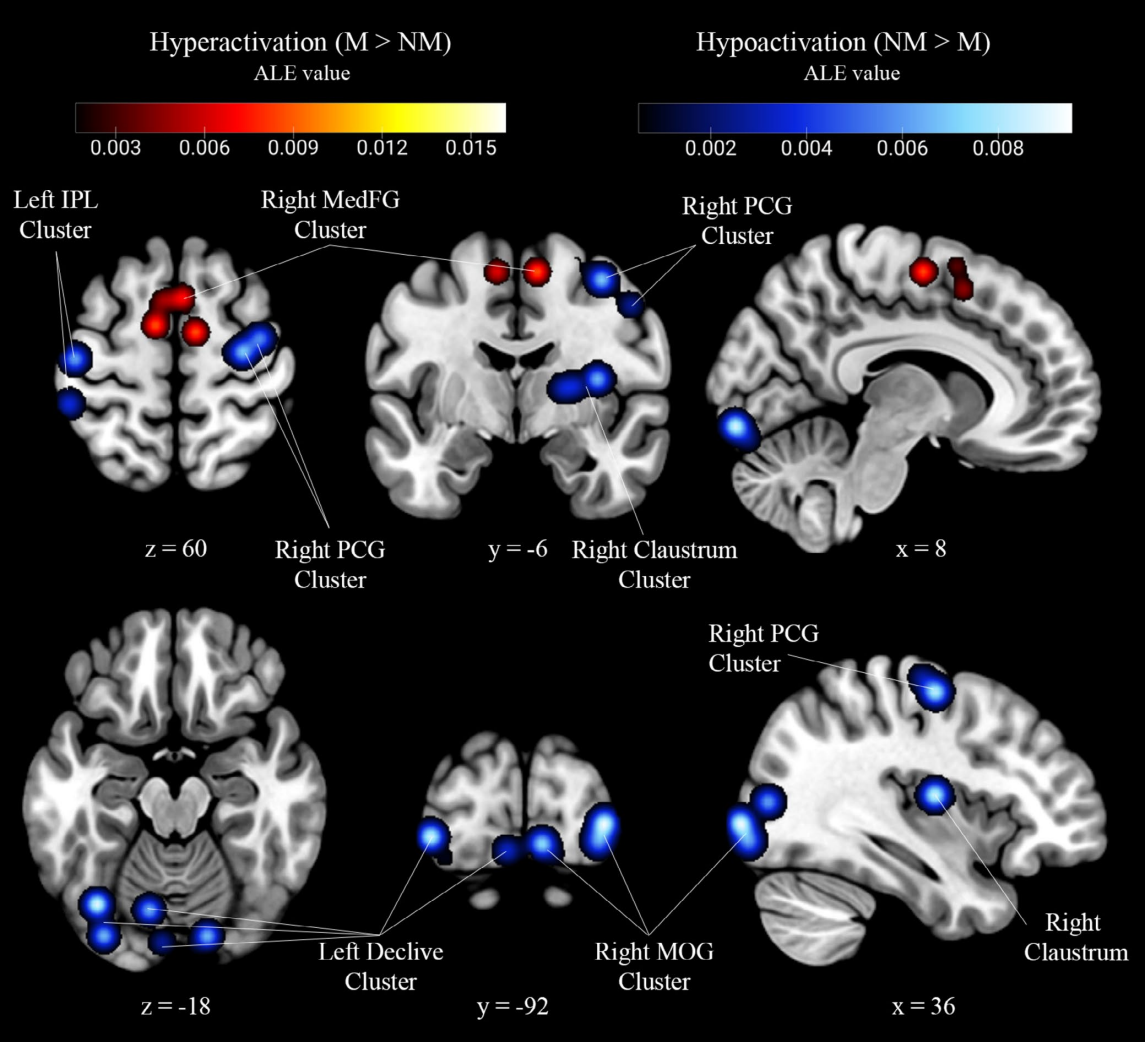
Hyperactivations (M > NM) and hypoactivations (NM > M) uncovered during ALE meta‐analysis. Red clusters represent M > NM contrasts. Blue clusters represent NM > M contrasts. Contrasts were computed with a cluster defining threshold of *p* < 0.05, FWE corrected. IPL, inferior parietal lobule; MedFG, medial frontal gyrus; MOG, middle occipital gyrus; PCG, precentral gyrus.

The M > NM contrast included data from 11 experimental contrasts (228 subjects, 78 Foci, 1 out of mask Foci). Results revealed significantly increased activation in musicians compared to non‐musicians in the right medial frontal gyrus (peak 8, −6, 60, volume = 7616 mm^3^, maximum ALE value = 0.0089). The NM > M contrast included data from 4 experimental contrasts (107 subjects, 38 foci, 1 out of mask foci). Results revealed significantly increased activation in non‐musicians compared to musicians across the right middle occipital gyrus (peak 36, −92, 0, volume = 23,432 mm^3^, maximum ALE value = 0.0095), the right precentral gyrus (peak 38, −8, 56, volume = 11,368 mm^3^, maximum ALE value = 0.0091), the left inferior parietal lobule (peak −54, −28, 42, volume = 10,904 mm^3^, maximum ALE value = 0.0088), the right claustrum (peak 36, −10, 12, volume = 9488 mm^3^, maximum ALE value = 0.0088), and the left declive (peak −36, −70, −18, volume = 8552 mm^3^, maximum ALE value = 0.0092). Figure 2 represents significant clusters uncovered from M > NM and NM > M ALE analyses. Table 1 outlines ALE meta‐analytic results for M > NM and NM > M contrasts at cluster level inference *p* < 0.05 (FWE).

**TABLE 1:** ALE meta‐analytic results for M > NM and NM > M contrasts at cluster‐level inference *p* < 0.05.

Musicians > non‐musicians									
Cluster number	Volume (mm^3^)	MNI coordinates			ALE	*p*	*Z*	Region	Brodmann area
		*x*	*y*	*z*					
#1	7616	8	−6	60	0.0089	3.88E‐4	3.36	R.Medial Frontal Gyrus	BA 6
		−8	−2	60	0.0087	5.10E‐04	3.29	L.Medial Frontal Gyrus	BA 6
		4	12	52	0.0086	5.34E‐04	3.27	R.Superior Frontal Gyrus	BA 6
		−6	8	56	0.0086	5.34E‐04	3.27	L.Medial Frontal Gyrus	BA 6
		2	8	62	0.0086	6.59E‐04	3.21	L.Medial Frontal Gyrus	BA 6

Non‐musicians > musicians									
Cluster number	Volume (mm^3^)	MNI coordinates			ALE	*p*	*Z*	Region	Brodmann area
		*x*	*y*	*z*					
#1	23,432	36	−92	0	0.0095	3.30E‐05	3.99	R.Middle Occipital Gyrus	BA 18
		10	−90	−10	0.0090	5.76E‐05	3.86	R.Lingual Gyrus	BA 18
		14	−84	−14	0.0090	5.97E‐05	3.85	R.Declive (Cerebellum)	—
		−6	−88	−12	0.0088	1.53E‐04	3.61	L.Declive (Cerebellum)	—
		−12	−72	−21	0.0088	1.53E‐04	3.61	L.Declive (Cerebellum)	—
		34	−82	8	0.0083	4.46E‐04	3.32	R.Middle Occipital Gyrus	BA 18
		−8	−104	−4	0.0083	4.46E‐04	3.32	L.Lingual Gyrus	BA 18
		34	−90	−6	0.0083	4.46E‐04	3.32	*R. inferior* Occipital Gyrus	BA 18
#2	11,368	38	−8	56	0.0091	5.24E‐05	3.88	R.Precentral Gyrus	BA 4
		30	−14	62	0.0087	1.68E‐04	3.59	R.Precentral Gyrus	BA 6
		50	−12	46	0.0085	3.75E‐04	3.37	R.Precentral Gyrus	BA 4
#3	10,904	−54	−28	42	0.0088	1.53E‐04	3.61	L.Inferior Parietal Lobule	BA 40
		−46	−18	56	0.0085	3.75E‐04	3.37	L.Inferior Postcentral Gyrus	BA 3
		−48	−38	56	0.0076	9.33E‐04	3.11	L.Inferior Parietal Lobule	BA 40
#4	9488	36	−10	12	0.0088	1.53E‐04	3.61	R.Claustrum	—
		26	0	8	0.0086	2.29E‐04	3.50	R.Lentiform Nucleus (Putamen)	—
		20	−12	8	0.0077	9.18E‐04	3.12	R.Thalamus (Ventral Lateral Nucleus)	—
#5	8552	−36	−70	−18	0.0092	4.30E‐05	3.93	L.Declive (Cerebellum)	—
		−34	−84	−16	0.0086	2.29E‐04	3.50	L.Declive (Cerebellum)	—
		−39	−93	−6	0.0086	2.29E‐04	3.50	L.Inferior Occipital Gyrus	BA 18

### 3.4 Meta‐Analytic Connectivity Modeling

**FIGURE 3:**
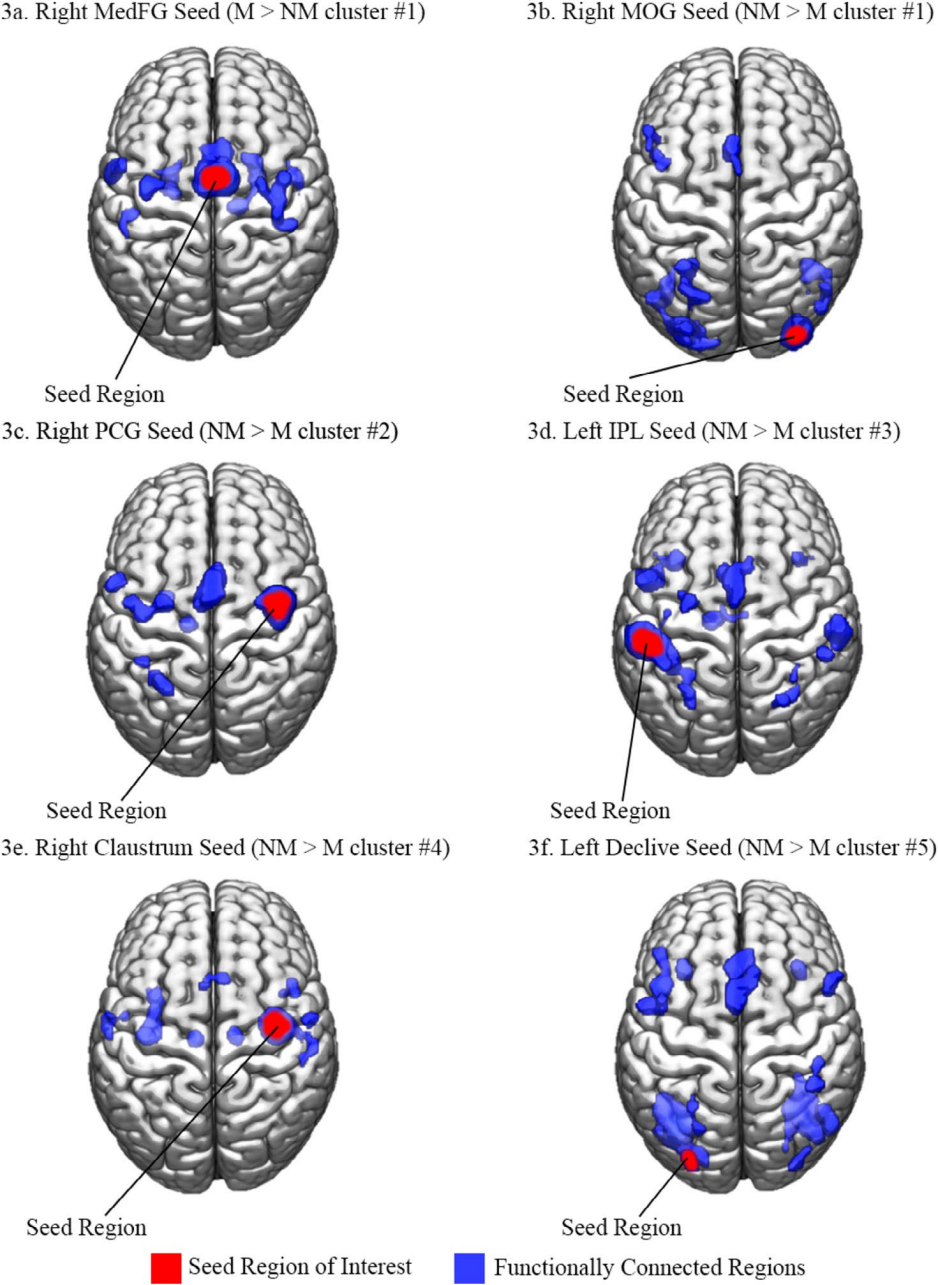
Meta‐analytic connectivity modeling for significant clusters identified in ALE analysis. Contrasts were computed with a cluster defining threshold of *p* < 0.001, FWE corrected. IPL, inferior parietal lobule; MedFG, medial frontal gyrus; MOG, middle occipital gyrus; PCG, precentral gyrus. Red areas highlight the seed region and do not represent differences in activation. Blue regions represent functionally connected regions (see Table S6). Variations in shading reflect anatomical depth, with lighter shades indicating deeper structures.

The right medial frontal gyrus ROI, identified with greater activation among musicians than non‐musicians in working memory tasks (M > NM Cluster #1), demonstrated coactivation (Based on 29 experiments with 426 subjects, 826 foci and 5 out of mask foci) with the right precentral gyrus, the right thalamus, the left lateral globus pallidus, the left precentral gyrus, the right insula and the left postcentral gyrus (Figure 3a).

The right middle occipital gyrus ROI, identified with greater activation among non‐musicians than musicians in working memory tasks (NM > M Cluster #1), demonstrated coactivation (Based on 28 experiments with 405 subjects, 665 Foci and 6 out of mask foci) with the left middle occipital gyrus, the left superior parietal lobule, the right culmen, the left medial frontal gyrus, and the left inferior frontal gyrus (Figure 3b).

The right precentral gyrus ROI, identified with greater activation among non‐musicians than musicians in working memory tasks (NM > M Cluster #2), demonstrated coactivation (Based on 31 experiments with 474 subjects, 542 Foci and 6 out of mask foci) with the left medial frontal gyrus, the left precentral gyrus, the left precuneus, the left precentral gyrus, the left putamen and the left thalamus (Figure 3c).

The left inferior parietal lobule ROI, identified with greater activation among non‐musicians than musicians in working memory tasks (NM > M Cluster #3), demonstrated coactivation (Based on 36 experiments with 450 subjects, 747 Foci and 14 out of mask foci) with the left cingulate gyrus, the left inferior frontal gyrus, the right postcentral gyrus, the left thalamus, the right superior parietal lobule, the left insula, the left middle frontal gyrus, and the right insula (Figure 3d).

The right claustrum ROI, identified with greater activation among non‐musicians than musicians in working memory tasks (NM > M Cluster #4), demonstrated coactivation (Based on 25 experiments with 380 subjects, 454 Foci and 4 out of mask foci) with the left claustrum, the left transverse temporal gyrus, the right cingulate gyrus, the right precentral gyrus, the right thalamus (medial dorsal nucleus), and the left thalamus (mammillary body) (Figure 3e).

The left declive ROI, identified with greater activation among non‐musicians than musicians in working memory tasks (NM > M Cluster #5), demonstrated coactivation (Based on 44 experiments with 700 subjects, 875 Foci and 7 out of mask foci) with the right declive, the left cingulate gyrus, the left inferior frontal gyrus, the right inferior parietal lobule, the left claustrum, the right inferior frontal gyrus, the right claustrum, the right middle occipital gyrus, and the left superior parietal lobule (Figure 3f). Figures 3a–e outline MACM results for each significant cluster (seed) identified during the ALE analysis. Table S6 outlines MACM ALE results for M > NM and NM > M contrasts at cluster level inference *p* < 0.001 (FWE).

### 3.5 Behavioral/Paradigm Analysis

The right medial frontal gyrus ROI, identified with greater activation among musicians than non‐musicians in working memory tasks (M > NM Cluster #1) was associated with action execution (*z* = 4.614), but not with any paradigm class. Clusters identified with greater activation among non‐musicians than musicians during working memory tasks (NM > M) also saw associations with a range of behavioral domains and paradigm classes. The right middle occipital gyrus ROI (NM > M Cluster #1) was not associated with any behavioral domain or paradigm class. The right precentral gyrus ROI (NM > M Cluster #2) was associated with action execution (*z* = 3.867), but not with any paradigm class. The left inferior parietal lobule ROI (NM > M Cluster # 3) was associated with action execution (*z* = 3.748), and somesthesis (*z* = 3.263). This ROI was also associated with the finger tapping paradigm class (*z* = 3.174). The right claustrum ROI (NM > M Cluster # 4) was associated with somesthesis (pain; *z* = 3.01) and the pain monitoring/discrimination paradigm class (*z* = 3.113). The left declive ROI (NM > M Cluster # 5) was associated with attention (*z* = 3.036), but not with any paradigm class.

### 3.6 Fail‐Safe N Analysis

FSN analysis for the M > NM contrast indicated that cluster #1 (right medial frontal gyrus, FSN = 4) met the lower bound robustness threshold. FSN analysis for the NM > M contrast showed that cluster #2 (right precentral gyrus; FSN = 5) and cluster #4 (subcortical cluster centred in the right claustrum; FSN = 5) met the lower bound robustness threshold. NM > M clusters #1 (right middle occipital gyrus), #3 (left inferior parietal lobule), and #5 (left cerebellar declive) fell below the lower bound threshold and were sensitive to the inclusion of null studies, suggesting potential susceptibility to publication bias. Accordingly, NM > M cluster #1, #3, and #5 should be interpreted with caution and would benefit from further support in future MRI investigations. Neither contrast exceeded the upper bound robustness threshold. Table 2 depicts the fail‐safe N robustness assessment, as well as contributing studies to each cluster and number of foci contributed.

**TABLE 2:** Fail‐safe *N* robustness assessment for significant M > NM and NM > M clusters.

Musicians > non‐musicians: 9 studies, 11 experiments, 228 subjects, 78 foci, minimum FSN = 3									
Cluster number	Volume (mm^3^)	MNI coordinates			Max ALE	Region	Contributing studies	Number of foci	FSN
		*x*	*y*	*z*					
#1	7616	8	−6	60	0.0089	R.Medial Frontal Gyrus	Pallesen et al. (2010)	2	4
							Pau et al. (2013)	2	
							Hoppe et al. (2014)	1	

Non‐musicians > musicians: 4 studies, 4 experiments, 107 subjects, 38 foci, minimum FSN = 1									
Cluster number	Volume (mm^3^)	MNI coordinates			Max ALE	Region	Contributing studies	Number of foci	FSN
		*x*	*y*	*z*					
#1	23,432	36	−92	0	0.0095	R.Middle Occipital Gyrus	Schmithorst and Holland 2004	1	< 1
							Pau et al. (2013)	3	
							Hoppe et al. (2014)	4	
#2	11,368	38	−8	56	0.0091	R.Precentral Gyrus	Schulze et al. (2011)	1	5
							Pau et al. (2013)	4	
#3	10,904	−54	−28	42	0.0088	L.Inferior Parietal Lobule	Schmithorst and Holland (2004)	1	< 1
							Pau et al. (2013)	3	
#4	9488	36	−10	12	0.0088	R.Claustrum	Schmithorst and Holland (2004)	1	5
							Pau et al. (2013)	2	
#5	8552	−36	−70	−18	0.0092	L.Declive (Cerebellum)	Schulze et al. (2011)	1	< 1
							Hoppe et al. (2014)	2	

Abbreviations: FSN, fail‐safe *N* analysis; *L*, left; Max ALE, maximum activation likelihood estimation in a cluster; *R*, right.

## 4 Discussion

**FIGURE 4:**
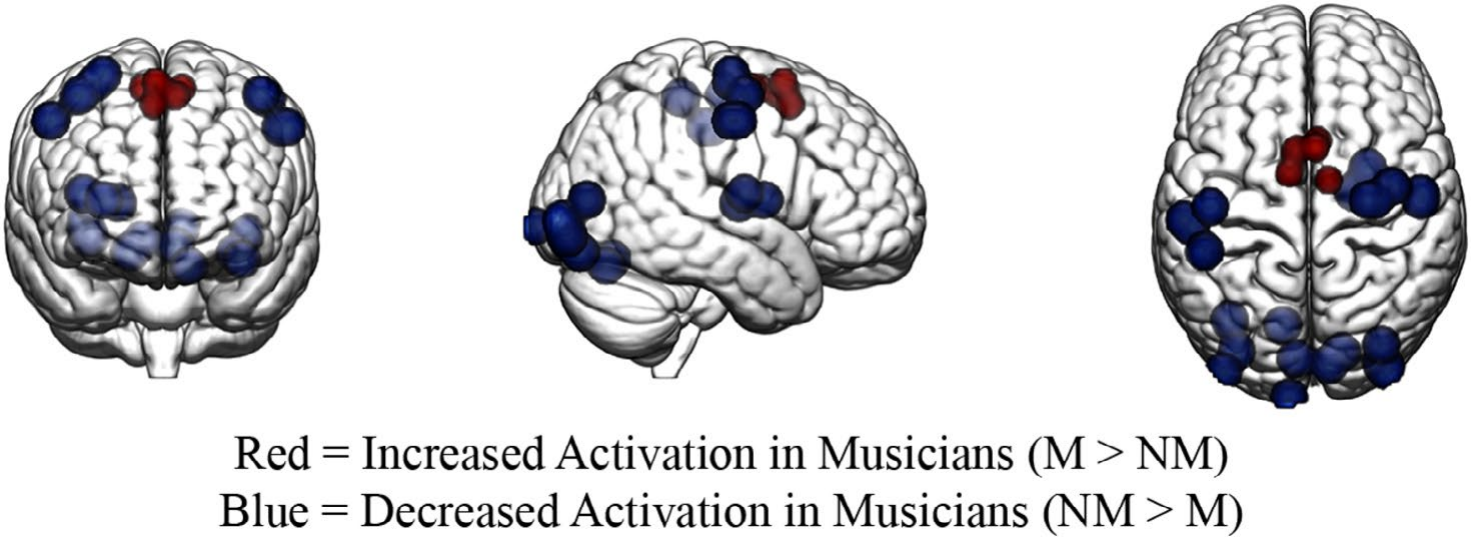
Summary of ALE findings. Figure outlines a 3D rendering of key M > NM and NM > M findings from the ALE analysis. Red clusters represent regional hyperactivation in musicians. Blue clusters represent regional hypoactivation in musicians. Variations in blue shading reflect anatomical depth, with lighter shades indicating deeper structures.

Expertise in musical activities requires individuals to integrate complex sensory, motor, and higher‐order cognitive processes to support both music perception, performance and the broader contextual factors associated with musical training. Our ALE meta‐analysis revealed convergences in the neural correlates of working memory function among musicians, suggesting consistent patterns of activation and deactivation during task performance compared to musically naïve individuals. The MACM analysis complemented these results, providing insights into network‐level working memory‐related co‐activation patterns among musicians. Finally, our behavioral and paradigm analyses characterized putative contributions of regions of interest to broader behavioral functions and common experimental designs, providing further context to ALE outcomes. Together, our findings support both hypotheses, suggesting that musicians engage both complementary pathways and more efficient core networks when performing working memory tasks. Figure 4 outlines key findings of the ALE meta‐analysis.

The first key finding was that increased activation in musicians relative to non‐musicians during working memory tasks was consistently localized to the right medial frontal gyrus. Given that working memory tasks generally recruit regions such as the prefrontal cortex, parietal cortex, medial temporal lobe, basal ganglia, and cerebellum, as well as some task specific areas (i.e., auditory areas during auditory working memory tasks: Eriksson et al. 2015; Nee et al. 2013), our results may suggest that musicians recruit outside of core working memory networks to support task performance.

In general, the medial frontal gyrus is thought to have associations with cognitive control, decision‐related processes (Talati and Hirsch 2005), performance monitoring, self‐regulation (van Noordt and Segalowitz 2012), and social cognition (Amodio and Frith 2006; Kim et al. 2020). The behavioral domain analysis also indicated associations between this region and action execution. Interestingly, there is some evidence to suggest that this region plays a role in implementing higher‐order executive functions (Frascarelli et al. 2015). Since working memory is often taxonomized as an executive function (Webb et al. 2018), our findings may suggest that musicians recruit executive function networks more broadly and actively than non‐musicians to support working memory performance. Similarly, the association between the medial frontal gyrus, social cognition, and motor execution may indicate that musicians better recruit socio‐emotional and motor‐cognitive networks to modify performance on working memory tasks.

MACM for this region of interest supports this idea, with our results revealing a cortico‐subcortical network of co‐activated brain regions typically associated with socio‐emotional and sensorimotor processing, including the precentral gyrus, postcentral gyrus, insula, and basal ganglia. Since musicians are reported to possess regional differences across these areas (Fiveash et al. 2023; Li et al. 2018; Olszewska et al. 2021), it is plausible that the medial frontal gyrus forms part of a broader network that primarily supports the socio‐emotional, sensorimotor, and executive functioning demands of musical activities (Miendlarzewska and Trost 2014; Wang 2022), whereas providing incidental benefits for generalized working memory function in musically trained individuals. Since it is inappropriate to infer causality from cross‐sectional findings, it is important to consider that these differences could either arise as a result of musical training, exist in musicians prior to measurement, or stem from a factor linked to both musicianship and neuroplastic adaptations in this region.

The second key finding was that several clusters showing greater activation in non‐musicians relative to musicians during working memory tasks were consistently localized to canonical working memory regions, including the parietal cortex (cluster with peak coordinates in the inferior parietal lobule), the cerebellum (cluster with peak coordinates in the declive), and subcortical areas such as the claustrum, thalamus, and basal ganglia (cluster with peak coordinates in the claustrum) (Eriksson et al. 2015). That said, two clusters associated with sensorimotor function (located in the precentral and middle occipital gyrus) were also identified with greater activation in non‐musicians than musicians during working memory tasks. Although not all studies reported on behavioral data related to working memory tasks (Schmithorst and Holland 2004), in general, musicians appeared to present with equivalent or enhanced performance relative to non‐musicians (Hoppe et al. 2014; Pau et al. 2013; Schulze et al. 2011). Together with prior meta‐analytic evidence suggesting musician advantage in working memory function (Talamini et al. 2017), activation clusters located in canonical working memory areas may imply that musicians make more efficient use of these regions, given that non‐musicians require higher activation to perform equal or worse behaviorally. In contrast, clusters in the precentral gyrus and middle occipital gyrus with little known connection to working memory function may instead reflect neural differences in sensorimotor processing between musicians and non‐musicians (Eriksson et al. 2015). Given motor associations of the precentral gyrus (Banker and Tadi 2025), activation in this cluster may reflect task‐specific motor demands among the included studies (Pau et al. 2013; Schulze et al. 2011), rather than reflecting a reliance of non‐musicians on motor pathways to execute working memory tasks. Similarly, activation in the middle occipital gyrus may reflect visual processing demands associated with in‐scanner working memory tasks, given the established role of this region in visual processing (Proverbio and Sanoubari 2024).

MACM findings generally support the ALE meta‐analysis. Coactivation with NM > M regions of interest was observed across key working memory areas like the parietal cortex, prefrontal cortex, and cerebellum, as well as subcortical regions like the basal ganglia and thalamus (Eriksson et al. 2015). Coactivation with limbic regions including the insula and cingulate gyrus was also observed. Meanwhile, the behavioral domain analysis revealed that activation clusters in non‐musicians were functionally associated with attention, action execution, and somesthesis, whereas the paradigm analysis revealed associations with interoceptive and finger‐tapping tasks. Collectively, these findings suggest that during working memory tasks, non‐musicians rely on more effortful engagement in regions associated with sensorimotor, interoceptive, and attentional processes, as well as networks that support higher‐order cognitive and socioemotional functions. Given that musical activities require substantial input from these domains (Miendlarzewska and Trost 2014; Wang 2022), it is possible that musicians implement these functions with greater neural efficiency, not only during musical performance but also during other cognitively demanding tasks. While our evidence is compatible with this interpretation, further research is required to better understand neural efficiency among musicians.

### 4.1 Critical Appraisal of the Included Studies

The studies included in the current review were screened to ensure methodological quality and suitability for coordinate‐based meta‐analysis. As is typical in neuroimaging research, sample sizes were modest, with most studies comparing fewer than 20 participants per group (Hay et al. 2022). However, the ALE framework explicitly accounts for this through its random‐effects model, where sample size determines the spatial uncertainty assigned to each experiment: larger samples contribute more spatially precise information, whereas smaller samples are modeled with broader kernels, reducing their influence on final convergence maps (Eickhoff et al. 2009; Müller et al. 2018). In addition, the included studies were characterized by relatively well‐balanced group sizes and clear operational definitions of musical expertise, supporting the interpretability of the resulting contrasts (see Section 3.1, “Characteristics of Included Studies”: Müller et al. 2018). Even so, these methodological advantages do not fully resolve the limitations imposed by small sample sizes. That is, meta‐analytic inferences are contingent on the robustness of the underlying data, and when primary studies are underpowered, the resulting convergence estimates may remain susceptible to noise. While fMRI research is inherently resource‐intensive and requires specialist expertise for acquisition and analysis, continued reliance on small samples constrains the reliability and generalisability of these studies and compromises meta‐analytic convergence patterns. The field's ongoing shift towards large‐scale open‐access datasets and multisite studies (i.e., Grassi et al. 2025) may provide a more stable basis for characterizing neuroplasticity associated with musical training and its relationship to working memory function. Accordingly, future research would benefit from leveraging such approaches.

Another methodological consideration is that a range of working memory paradigms were employed across studies, including verbal, tonal, and visuospatial encoding/retrieval tasks, n‐back, mental rotation, and mental arithmetic (For task details, see Table S4). Although convergent activation across diverse paradigms could indeed reflect domain‐general mechanisms underlying working memory in musicians, such variability also means that the current results cannot be unambiguously interpreted as evidence for a single generalized working memory mechanism. This is particularly important considering the evidenced dissociation between musician advantage across working memory domains (Talamini et al. 2017). Additionally, overlapping mechanisms implemented during different working memory tasks (e.g., sustained attention, executive control, or sensorimotor processing) introduce construct ambiguity, such that identified convergence could instead represent neural differences associated with broader higher‐order cognitive processes. Although most working memory paradigms are considered to adequately index the construct, these factors may nonetheless act as potential confounds in the current data. Addressing this limitation will require greater standardization of working memory paradigms in future research, where tasks that isolate specific component processes of working memory function are employed across similar stimulus modalities (i.e., musical, verbal, or visuospatial modalities). A greater emphasis on pre‐registration of whole‐brain analyses may support such standardization by facilitating cross‐institutional coordination and mitigating bias introduced by selective reporting of neuroimaging results. Longitudinal approaches may further help to characterize and dissociate cognitive mechanisms underlying working memory in musicians, particularly in designs incorporating multiple tasks targeting distinct domains.

## 5 Conclusion

By integrating findings from across neuroimaging experiments, the present study outlines consistent patterns of brain activation and deactivation in musicians compared to non‐musicians during working memory tasks. Complementary findings from MACM and the behavioral/paradigm analysis contextualized expertise‐related regions and “networks” of interest in musicians, further strengthening the findings. However, several limitations in the present study should be considered. First, the constrained sample sizes and limited number of studies included in the final analysis reduced the strength of our findings (Müller et al. 2018). To an extent, this limitation is reflected in the instability of clusters that did not survive the fail‐safe *N* robustness check; accordingly, results potentially influenced by the file‐drawer effect should be interpreted with caution. Given the extant literature in this area, our investigation was restricted by the number of studies available for inclusion. Furthermore, our approach prioritized homogeneity between paradigm classes and explicit links to working memory function in experimental contrasts. Future research could benefit from expanding the scope of this study and incorporating a variety of behavioral tasks to determine how musicians' brains process different cognitive functions. Additionally, to maximize coverage of the musician literature, our search strategy combined standard musician‐related terms (e.g., musician, musical training) with common instrument‐specific descriptors (e.g., pianist, violinist, guitarist, drummer, vocalist). Although less common instrument‐specific terminology may represent a residual source of missed records, the inclusion of these terms did not yield any additional studies meeting our criteria, suggesting that our findings are unlikely to have been materially affected by this limitation.

Given the cross‐sectional design, there are also limits to the interpretation and generalizability of our results. For instance, while training‐related plasticity may account for the neural activation patterns observed among musicians, it is possible that baseline predispositions (i.e., genetic factors: Mosing et al. 2014), or other lifestyle factors associated with musicianship and neuroplasticity (i.e., socioeconomic status or level of education) may better explain neurocognitive differences between groups. As such, future research should consider longitudinal designs or randomized controlled trials to mitigate the effect of confounding variables and explore whether neural adaptations are stable over time or dissipate with age (Salthouse 2006; Tucker‐Drob 2019). Finally, to expand upon the speculated socio‐emotional benefits of musical activities, it is crucial for future research to differentiate between neural adaptations arising from training‐related cognitive demands and those linked to the various social, emotional, and motivational factors that are contextually associated with musical training. As such, examining how varying levels of music engagement (i.e., casual vs. professional) influence neurocognitive function may help to disentangle expertise‐related plasticity from broader effects of music participation.

To conclude, the findings from this study explore the relationship between musicianship and the neural implementation of working memory. Our analysis identified complementary regions that musicians engage during working memory tasks, while highlighting regions that may be more efficient in musicians. Importantly, these findings outline the potential of lifelong music engagement not only as a means of cognitive enrichment, but as a modifiable lifestyle factor that may help to build and sustain cognitive functions across the lifespan.

## Author Contributions

**Lee Wolff:** conceptualization (equal), data curation (lead), formal analysis (lead), investigation (lead), methodology (lead), project administration (equal), validation (lead), visualization (lead), writing – original draft (lead), writing – review and editing (equal). **Yixue Quan:** conceptualization (equal), formal analysis (supporting), investigation (equal), methodology (equal), writing – review and editing (equal). **William Forde Thompson:** conceptualization (equal), funding acquisition (lead), methodology (equal), project administration (equal), supervision (lead), writing – review and editing (equal). **Oliver Baumann:** conceptualization (equal), formal analysis (supporting), investigation (supporting), methodology (equal), project administration (equal), supervision (lead), writing – review and editing (equal).

## Funding

This research was supported by a Discovery Project grant awarded to W.F.T. by the Australian Research Council (DP210101247).

## Conflicts of Interest

The authors declare no conflicts of interest.

## Related WIREs Articles

Music perception and cognition: development, neural basis, and rehabilitative use of music.

A systematic review and meta‐analysis of memory‐guided attention: Frontal and parietal activation suggests involvement of fronto‐parietal networks.

## Supporting information


**Table S1:** Search terms and number of items identified by database on the 21/01/2026.
**Table S2:** Eligibility criteria.
**Table S3:** List of excluded studies and reasons for exclusion.
**Table S4:** Characteristics of studies included in meta‐analysis.
**Table S5:** Characteristics of MRI acquisition for studies included in meta‐analysis.
**Table S6:** Meta‐analytic connectivity modeling (MACM) results for M > NM and NM > M contrasts at cluster‐level inference *p* < 0.05.

## Data Availability

The data that support the findings of this study are openly available in OSF at 10.17605/OSF.IO/4SHXN, reference number 4SHXN.
