# Supplementary material for: Functional Neural Architecture of Working Memory in Musicians: An ALE Meta‐Analysis and Review
Source: Wiley Interdiscip Rev Cogn Sci. 2026 Jun 14;17(3):e70036. doi: 10.1002/wcs.70036 (PMC13265137; doi:10.1002/wcs.70036)
Supplement: Supplementary file 1 — Table S1: Search terms and number of items identified by database on the 21/01/2026. Table S2: Eligibility criteria. Table S3: List of excluded studies and reasons for exclusion. Table S4: Characteristics of studies included in meta‐analysis. Table S5: Characteristics of MRI acquisition for studies included in meta‐analysis. Table S6: Meta‐analytic connectivity modeling (MACM) results for M > NM and NM > M contrasts at cluster‐level inference p < 0.05. [file WCS-17-e70036-s001.docx]

**Functional Neural Architecture of Working Memory in Musicians: An ALE Meta-Analysis and Review**

**Supplementary Materials**

Lee Wolff^1^*, Yixue Quan^2^, William F, Thompson, PhD^1,2^, and Oliver Baumann, PhD^1^

^1^Bond University, Gold Coast, QLD, Australia

^2^Macquarie University, Sydney, NSW, Australia

**Corresponding Author:**

*Lee Wolff, Faculty of Society and Design, Bond University, 14 University Drive, ROBINA, Gold Coast, Queensland, 4226, Australia

Email: lewolff@bond.edu.au

ORCID ID: https://orcid.org/0009-0009-7817-934X**Supplementary table 1.**

*Search terms and number of items identified by database on the 21/01/2026*

| **Database** | **Search String** |
| --- | --- |
| **Pubmed**  Number of items identified: 479 | ((Musician* OR "Musical Expert*" OR "Music Expert*" OR "Musical Train*" OR "Music Train*" OR "Musically Trained" OR Pianist* OR Violinist* OR Guitarist* OR Drummer* OR Vocalist* ) AND (Imaging OR Neuroimaging OR "Functional neuroimaging" OR fMRI OR "Functional Magnetic Resonance Imaging" OR PET OR "Positron Emission Tomography" ) AND ("Working Memory" OR Memory OR Cognit* OR "Executive function*" )) |
| **Cochrane library**  Number of items identified: 19 | ((Musician* OR ("Musical" NEXT Expert*) OR ("Music" NEXT Expert*) OR ("Musical" NEXT Train*) OR ("Music" NEXT Train*) OR "Musically Trained" OR Pianist* OR Violinist* OR Guitarist* OR Drummer* OR Vocalist*) AND (Imaging OR Neuroimaging OR "Functional neuroimaging" OR fMRI OR "Functional Magnetic Resonance Imaging" OR PET OR "Positron Emission Tomography") AND ("Working Memory" OR Memory OR Cognit* OR ("Executive" NEXT function*))) |
| **Embase (Elsevier)**  Number of items identified: 526 | ((Musician* OR 'Musical Expert*' OR 'Music Expert*' OR 'Musical Train*' OR 'Music Train*' OR 'Musically Trained' OR Pianist* OR Violinist* OR Guitarist* OR Drummer* OR Vocalist*) AND (Imaging OR Neuroimaging OR 'Functional neuroimaging' OR fMRI OR 'Functional Magnetic Resonance Imaging' OR PET OR 'Positron Emission Tomography') AND ('Working Memory' OR Memory OR Cognit* OR 'Executive function*')) |
| **Web of Science**  Number of items identified: 594 | ((Musician* OR "Musical Expert*" OR "Music Expert*" OR "Musical Train*" OR "Music Train*" OR "Musically Trained" OR Pianist* OR Violinist* OR Guitarist* OR Drummer* OR Vocalist*) AND (Imaging OR Neuroimaging OR "Functional neuroimaging" OR fMRI OR "Functional Magnetic Resonance Imaging" OR PET OR "Positron Emission Tomography") AND ("Working Memory" OR Memory OR Cognit* OR "Executive function*")) |
| **Scopus**  Number of items identified: 371 | ((Musician* OR "Musical Expert*" OR "Music Expert*" OR "Musical Train*" OR "Music Train*" OR "Musically Trained" OR Pianist* OR Violinist* OR Guitarist* OR Drummer* OR Vocalist*) AND (Imaging OR Neuroimaging OR "Functional neuroimaging" OR fMRI OR "Functional Magnetic Resonance Imaging" OR PET OR "Positron Emission Tomography") AND ("Working Memory" OR Memory OR Cognit* OR "Executive function*")) |
| **Psychinfo (Ovid)**  Number of items identified: 209 | ((Musician* OR "Musical Expert*" OR "Music Expert*" OR "Musical Train*" OR "Music Train*" OR "Musically Trained" OR Pianist* OR Violinist* OR Guitarist* OR Drummer* OR Vocalist*) AND (Imaging OR Neuroimaging OR "Functional neuroimaging" OR fMRI OR "Functional Magnetic Resonance Imaging" OR PET OR "Positron Emission Tomography") AND ("Working Memory" OR Memory OR Cognit* OR "Executive function*")) |
| **Medline (Ovid)**  Number of items identified: 236 | ((Musician* OR "Musical Expert*" OR "Music Expert*" OR "Musical Train*" OR "Music Train*" OR "Musically Trained" OR Pianist* OR Violinist* OR Guitarist* OR Drummer* OR Vocalist*) AND (Imaging OR Neuroimaging OR "Functional neuroimaging" OR fMRI OR "Functional Magnetic Resonance Imaging" OR PET OR "Positron Emission Tomography") AND ("Working Memory" OR Memory OR Cognit* OR "Executive function*")) |

**Supplementary table 2.**

*Eligibility criteria*

| **PICOS** | **Inclusion criteria** | **Exclusion criteria.** |
| --- | --- | --- |
| Population | Healthy adult participants (aged 18 and over) with and without musical training who undertook in-scanner working memory tasks during fMRI. | Clinical populations; participants under the age of 18. |
| Intervention | Extensive formal musical training. Participants with musical training must be considered ‘expert’ musicians. | Contrasts between special groups of musicians (e.g., absolute pitch vs. relative pitch musicians). |
| Comparison | No formal musical training (outside of obligatory school-based music classes). | Comparator groups with expertise in non-musical skills. |
| Outcome | Differences in task-based functional hemodynamic activity related to working memory performance. Data must be reported as between-group contrasts comparing neural responses in musicians and non-musicians (e.g., M > NM or NM > M) using MNI or Talairach stereotactic coordinates. | Studies reporting structural neuroimaging data (e.g., MRI, DTI); neuroimaging modalities incompatible with task-based fMRI or PET (e.g., EEG, MEG, SPECT, fNIRS); resting-state fMRI studies; studies using only functional connectivity or region-of-interest analyses; studies not reporting MNI or Talairach coordinates. |
| Study design | Cross-sectional neuroimaging studies published in full-length, peer-reviewed English-language journals. | Randomized controlled trials, longitudinal studies, cohort studies, case reports, protocols, review articles, book chapters, dissertations, conference abstracts, and animal studies. |

**Supplementary table 3.**

*List of excluded studies and reasons for exclusion*

| **Excluded studies** | **Exclusion reason** |
| --- | --- |
| Alain, C., Khatamian, Y., He, Y., Lee, Y., Moreno, S., Leung, A. W. S., & Bialystok, E. (2018). Different neural activities support auditory working memory in musicians and bilinguals. Annals of the New York Academy of Sciences. https://doi.org/10.1111/nyas.13717 | Results were presented across musicians, non-musicians, and bilinguals, without a direct pairwise comparison between musicians and non-musicians. |
| Bastepe-Gray, S. E., Acer, N., Gumus, K. Z., Gray, J. F., & Degirmencioglu, L. (2020). Not all imagery is created equal: A functional Magnetic resonance imaging study of internally driven and symbol driven musical performance imagery. Journal of Chemical Neuroanatomy, 104, 101748. https://doi.org/10.1016/j.jchemneu.2020.101748 | The experiment used a within-subjects design. |
| Bengtsson, S. L., Csíkszentmihályi, M., & Ullén, F. (2007). Cortical regions involved in the generation of musical structures during improvisation in pianists. Journal of Cognitive Neuroscience, 19(5), 830–842. Embase. https://doi.org/10.1162/jocn.2007.19.5.830 | The experiment used a within-subjects design. |
| Berrocal, J. (2013). MUSIC AND NEUROSCIENCE: One more step in the knowledge of the human being. ARTSEDUCA, 4. | The full article could not be retrieved. The author and journal were contacted, but no response was received. |
| Brown, S., & Martinez, M. J. (2007). Activation of premotor vocal areas during musical discrimination. Brain and Cognition, 63(1), 59–69. https://doi.org/10.1016/j.bandc.2006.08.006 | The experiment was a within-subjects design. |
| Brown, R. M., & Penhune, V. B. (2018). Efficacy of auditory versus motor learning for skilled and novice performers. Journal of Cognitive Neuroscience, 30(11), 1657–1682. APA PsycInfo <2018>. https://doi.org/10.1162/jocn_a_01309 | The experiment used a within-subjects design. |
| Burunat, I., Alluri, V., Toiviainen, P., Numminen, J., & Brattico, E. (2014). Dynamics of brain activity underlying working memory for music in a naturalistic condition. Cortex; a Journal Devoted to the Study of the Nervous System and Behavior, 57, 254–269. https://doi.org/10.1016/j.cortex.2014.04.012 | The experiment used a within-subjects design. |
| Cheung, V. K. M., Meyer, L., Friederici, A. D., & Koelsch, S. (2018). The right inferior frontal gyrus processes nested non-local dependencies in music. Scientific Reports, 8(1), 3822. Medline. https://doi.org/10.1038/s41598-018-22144-9 | The experiment used a within-subjects design. |
| de Manzano, Ö., & Ullén, F. (2012). Goal-independent mechanisms for free response generation: Creative and pseudo-random performance share neural substrates. NeuroImage, 59(1), 772–780. https://doi.org/10.1016/j.neuroimage.2011.07.016 | The outcome measure primarily assessed neural correlates of creative and pseudo-random improvisation, rather than working memory. |
| Ellis, R. J., Norton, A. C., Overy, K., Winner, E., Alsop, D. C., & Schlaug, G. (2012). Differentiating maturational and training influences on fMRI activation during music processing. NeuroImage, 60(3), 1902–1912. https://doi.org/10.1016/j.neuroimage.2012.01.138 | Musical training was used as a regressor, and results did not include a direct pairwise comparison between musicians and non-musicians. |
| Foster, N. E. V., Halpern, A. R., & Zatorre, R. J. (2013). Common parietal activation in musical mental transformations across pitch and time. NeuroImage, 75, 27–35. https://doi.org/10.1016/j.neuroimage.2013.02.044 | The experiment used a within-subjects design. |
| Foster, N. E. V., & Zatorre, R. J. (2010). A role for the intraparietal sulcus in transforming musical pitch information. Cerebral Cortex (New York, N.Y.: 1991), 20(6), 1350–1359. https://doi.org/10.1093/cercor/bhp199 | The experiment only reported within-subjects outcomes. |
| Gaab, N., & Schlaug, G. (2003a). Musicians Differ from Nonmusicians in Brain Activation despite Performance Matching. Annals of the New York Academy of Sciences, 999(1), 385–388. https://doi.org/10.1196/annals.1284.048 | No MNI or Talairach coordinates were reported. The authors were contacted, but no response was received. |
| Gaab, N., & Schlaug, G. (2003b). The effect of musicianship on pitch memory in performance matched groups. Neuroreport, 14(18), 2291–2295. https://doi.org/10.1097/00001756-200312190-00001 | No MNI or Talairach coordinates were reported. The authors were contacted, but no response was received. |
| Gaab, N., Tallal, P., Kim, H., Lakshminarayanan, K., Archie, J. J., Glover, G. H., & Gabrieli, J. D. E. (2005). Neural correlates of rapid spectrotemporal processing in musicians and nonmusicians. Annals of the New York Academy of Sciences, 1060, 82–88. https://doi.org/10.1196/annals.1360.040 | No MNI or Talairach coordinates were reported. The authors were contacted, but no response was received. |
| Gagnepain, P., Fauvel, B., Desgranges, B., Gaubert, M., Viader, F., Eustache, F., Groussard, M., & Platel, H. (2017). Musical Expertise Increases Top-Down Modulation Over Hippocampal Activation during Familiarity Decisions. Frontiers in Human Neuroscience, 11, 472. https://doi.org/10.3389/fnhum.2017.00472 | The experiment utilised a region-of-interest analysis. |
| Guo, X., Yamashita, M., Suzuki, M., Ohsawa, C., Asano, K., Abe, N., Soshi, T., & Sekiyama, K. (2021). Musical instrument training program improves verbal memory and neural efficiency in novice older adults. Human Brain Mapping, 42(5), 1359–1375. https://doi.org/10.1002/hbm.25298 | The results did not include a direct pairwise comparison between musicians and non-musicians during task-related activation. |
| Grahn, J. A., & Schuit, D. (2012). Individual differences in rhythmic ability: Behavioral and neuroimaging investigations. Special Issue: Neurosciences and Music, 22(2), 105–121. APA PsycInfo <2012>. https://doi.org/10.1037/a0031188 | The sample was mixed and included children. |
| Groussard, M., La Joie, R., Rauchs, G., Landeau, B., Chételat, G., Viader, F., Desgranges, B., Eustache, F., & Platel, H. (2010). When music and long-term memory interact: Effects of musical expertise on functional and structural plasticity in the hippocampus. PloS One, 5(10), e13225. https://doi.org/10.1371/journal.pone.0013225 | The outcome variable primarily measured long-term memory and familiarity with excerpt melodies, rather than working memory. |
| James, C. E., Altenmüller, E., Kliegel, M., Krüger, T. H. C., Van De Ville, D., Worschech, F., Abdili, L., Scholz, D. S., Jünemann, K., Hering, A., Grouiller, F., Sinke, C., & Marie, D. (2020). Train the brain with music (TBM): Brain plasticity and cognitive benefits induced by musical training in elderly people in Germany and Switzerland, a study protocol for an RCT comparing musical instrumental practice to sensitization to music. BMC Geriatrics, 20(1), 418. https://doi.org/10.1186/s12877-020-01761-y | Study protocol for an RCT with no original data embedded. |
| Koelsch, S., Fritz, T., Schulze, K., Alsop, D., & Schlaug, G. (2005). Adults and children processing music: An fMRI study. NeuroImage, 25(4), 1068–1076. https://doi.org/10.1016/j.neuroimage.2004.12.050 | The sample was mixed and included children. No significant between-group differences were reported. |
| Landau, S. M. (2005). Practice and neural efficiency: An fMRI study of the influence of expertise on working memory processes. Dissertation Abstracts International: Section B: The Sciences and Engineering, 66(2-B), 743. | A dissertation submitted for the degree of Doctor of Philosophy. All grey literature was excluded from use in the present study. |
| McDermott, A. (2021). At the nexus of music and medicine, some see treatments for disease. Proceedings of the National Academy of Sciences of the United States of America, 118(4). Embase. https://doi.org/10.1073/pnas.2025750118 | Review article with no original data. |
| Oechslin, M. S., Van De Ville, D., Lazeyras, F., Hauert, C.-A., & James, C. E. (2013). Degree of musical expertise modulates higher order brain functioning. Cerebral Cortex (New York, N.Y.: 1991), 23(9), 2213–2224. https://doi.org/10.1093/cercor/bhs206 | The results did not include a direct pairwise comparison between musicians and non-musicians. |
| Pecenka, N., Engel, A., & Keller, P. (2013). Neural correlates of auditory temporal predictions during sensorimotor synchronization. Frontiers in Human Neuroscience, 7. https://www.frontiersin.org/articles/10.3389/fnhum.2013.00380 | The experiment used a within-subjects design. |
| Sakreida, K., Higuchi, S., Di Dio, C., Ziessler, M., Turgeon, M., Roberts, N., & Vogt, S. (2018). Cognitive Control Structures in the Imitation Learning of Spatial Sequences and Rhythms-An fMRI Study. Cerebral Cortex (New York, N.Y. : 1991), 28(3), 907–923. https://doi.org/10.1093/cercor/bhw414 | The outcome variable primarily assessed cognitive control, rather than working memory. |
| Wang, X., Yamashita, M., Guo, X., Stiernman, L., Kakihara, M., Abe, N., & Sekiyama, K. (2025). Never too late to start musical instrument training: Effects on working memory and subcortical preservation in healthy older adults across 4 years. Imaging Neuroscience, 3, IMAG.a.48. https://doi.org/10.1162/IMAG.a.48 | The experiment employed a functional connectivity analysis, providing data incompatible with ALE format. |
| Zatorre, R. J., Halpern, A. R., & Bouffard, M. (2010). Mental reversal of imagined melodies: A role for the posterior parietal cortex. Journal of Cognitive Neuroscience, 22(4), 775–789. https://doi.org/10.1162/jocn.2009.21239 | The experiment used a within-subjects design. |

**Supplementary table 4.**

*Characteristics of studies included in meta-analysis.*

| Author (Year) | Sample size (Gender ratio) | Age (years) | Sample Type & Experience | Task & contrast |
| --- | --- | --- | --- | --- |
| Hoppe (2014) | Musicians: 15 (7M, 8F)  Non-musicians: 15 (7M, 8F) | Musicians: M = 25.6, SD = 2.7  Non-musicians: M = 25.9, SD = 2.1 | Musicians were either professional singers (n=1), singing students at local conservatory (n=7) or ambitioned amateurs with prior lessons, regular practice and experienced in high-level choral performances (n = 7). Control group had no formal musical education. | Subjects were required to perform delayed matching-to-sample tasks on visually or auditorily presented sample and probe sequences. The duration of the retention interval was 10 s. Sequence 1 was simple visuospatial matching (VV), sequence 2 was a tonal matching task (TT) and sequence 3 was a visuotonal matching task (VT). Between-groups contrasts (M>NM and NM>M) contrasts were provided for VT>VV and VT>TT activations. |
| Huang (2010) | Musicians: 10 (10F)  Non-musicians: 10 (10F) | Musicians: Range = 20 – 23, M = 21.3, SD = 1.1  Non-musicians: Range = 20-23, M = 21.6, SD = 1.3 | All musicians were pianists who had started piano before the age of 7, continued for a period of more than 8 years, and passed a national piano test. Control group had no formal musical education. | Subjects were required to conduct verbal encoding, verbal retrieval and a pitch judgement control task. Encoding and retrieval tasks required participants to listen, encode (40s block) and recall (30s block) a list of words presented auditorily. The pitch judgement control task required the comparison of tone pairs (high-low vs low-high) during a 20s block. Between-groups contrasts (M>NM) were provided for performance during retrieval block relative to the control task. |
| Pallesen (2010) | Musicians: 11 (2M, 9F)  Non-musicians: 10 (5M, 5F) | Musicians: Range = 21-34, M = 28  Non-musicians: Range = 22-31, M = 25 | Musicians were either students or graduates of the Sibelius Music Academy in Finland. Non-musicians had only completed obligatory musical training in primary school. | Subjects were required to memorize octave chords that were either major, minor or dissonant according to western tonal music theory. The experimental conditions were either an easy 1-back task (1B) or difficult 2-back (2B) task, or a passive listening control without cognitive evaluation (all during 60s blocks). Between-groups contrasts (M>NM) were provided for 2B performance relative to 1B performance. |
| Pau (2013) | Musicians: 14 (8M, 6F)  Non-musicians: 15 (9M, 6F) | Musicians: M = 24.0, SD = 3.11  Non-musicians: M = 25.4, SD = 1.18 | Musicians were pianists who started practicing the piano at an average age of M = 8.43, SD = 2.98. Musicians had played on average for M = 11.36 years SD = 4.58 years. All musicians reported an average of M = 6.61 hours, SD = 9.3 hours of piano practice per week over the previous 3 months. Musically naïve participants had not received formal musical training outside of obligatory school-based music training. | Subjects, after familiarizing themselves with tone-to-key assignments of eight keys were required to encode visually presented finger sequences (24s block) and then retrieve/ replay sequences with and without auditory feedback (12s). Between-groups contrasts were presented in two separate tables. Table 1 presented M>NM contrast during the encoding phase of the trial. Table 2 presented NM>M contrast during the retrieval phase with auditory feedback. Table 3 was a region of interest contrast on single participant groups. |
| Schmithorst (2004) | Musicians: 7  Non-Musicians: 8  Gender split across whole sample: 11M, 4F | Age across whole sample: M = 37.8, SD = 15.2 | Musicians had studied either a musical instrument or voice since early childhood (8 years old or younger). These participants engaged in study continuously since childhood and throughout adolescence. Non-musicians had no formal musical training. | Subjects were presented with three mathematical fraction problems (either addition or subtraction) to perform mentally. They were provided with 10s to solve each problem. The control condition presented numbers in the same position as mathematical problems for 10s, however, divisor bars and plus/ minus signs were absent. Subjects were given a 3s visual cue to indicate ‘active’ or ‘rest’ between conditions. Between-groups contrasts (M>NM and NM>M) were provided for performance during mental mathematics tasks. |
| Schulze (2010) | Musicians: 16 (9M, 7F)  Non-musicians: 17 (9M, 8F) | Musicians: Range = 20-27, M = 23.5, SEM = 0.61  Non-musicians Range = 21-29, M = 25.47 | Musicians had studied an instrument at the University of Music and Theatre Mendelsohn Bartholdy in Leipzig. Musicians either studied the piano (n = 8), a woodwind instrument (n = 4), a string instrument (n = 3) or a brass instrument (n = 1). Non-musicians had received no formal musical education aside from school-based learning. | Subjects were required to listen to sine wave tones for 400ms intervals. Stimuli were either tonal (in one tonal key, with three notes belonging to a triad) or atonal (neither triad nor key) sequences. Subjects were presented with sequences and then required to rehearse sequences for 4200ms. At the end of this period, a test stimulus tone was presented, and subjects were asked to determine whether this tone had been presented in the prior tone sequence. Between-groups contrasts (M>NM) were provided for tonal vs atonal processing during the retrieval phase. |
| Schulze (2011) | Musicians: 16 (9M, 7F)  Non-musicians: 17 (9M, 8F) | Musicians: Range = 20-27, M = 23.5, SEM = 0.63  Non-musicians Range = 21-29, M = 25.47 | Musicians had studied an instrument at the University of Music and Theatre Mendelsohn Bartholdy in Leipzig. Musicians either studied the piano (n = 8), a woodwind instrument (n = 4), a string instrument (n = 3) or a brass instrument (n = 1). Non-musicians had received no formal musical education aside from school-based learning. | Subjects were required to listen to auditory stimuli which consisted of a spoken syllable and a simultaneously presented sine wave tone of the same loudness. Five stimuli were presented in a sequence, with each stimulus possessing a duration of 400ms. Including 150ms breaks, the total sequence length was 2600ms. Following the listening block, participants were required to rehearse either the tonal or verbal sequence (depending on the condition). After this, participants were required to listen to a single test auditory stimulus (simultaneous syllable and tone) and determine whether the syllable in the verbal condition, or tone in the tonal condition, were present during the sample stimuli sequence. The control condition was a pink noise passive listening block to control for auditory perception. Between-groups contrasts (M>NM and NM>M) were presented for both tonal and verbal conditions relative to the non-rehearsal control condition. |
| Sluming (2007) | Musicians: 10 (10M)  Non-musicians: 10 (10M) | Musicians: M = 42.2, SD = 10.1  Non-musicians M = 40.1, SD = 11.8 | Musicians were all professional instrumentalists in the Liverpool philharmonic orchestra. Musicians either played violin (n = 7), cello (n = 2), or double bass (n = 1). The control group was matched for handedness, age and verbal intelligence and had no formal musical training. | Subjects were required to perform a 3D mental rotation task. Stimuli were pairs of three-dimensional perspective drawings that consisted of 10 cubes in chiral patterns. Drawings were displayed side by side on the screen, with one vertically oriented, while the comparison image could be presented at angular increments from the vertical position either clockwise or counterclockwise. Participants were required to determine whether the rotated image was the same or different from the original image. A control task, where subjects were to judge same or different 2D shapes, was implemented to control for normal visual processing. Between-groups contrasts (M>NM) were presented during 3DMR relative to the 2D control condition. |
| Yamashita (2022) | Musicians: 30 (11M, 19F)  Non-musicians: 30 (13M, 17F) | Musicians: M = 70.8, SD = 4.0  Non-musicians M = 71.4, SD = 4.6 | All musicians commenced musical instrument training between the ages of 3 and 16 years (M = 8.6). They had at least 22 years of experience playing a musical instrument on a regular basis (M = 52.7, range 22–70) and had been playing a musical instrument for more than 10 years at the time the study was conducted (M= 46.4 years, range 10–70). All musicians were actively playing an instrument at the time of the study. The instruments included piano, violin, cello, electric guitar, mandolin, wood bass, ukulele, viola, electronic clarinet, and alto saxophone. Controls had received less than three years of formal training or no training. | Subjects were scanned during three conditions: a 1-back working memory task for melodies, a 0-back hearing a melody task and rest. In the 1-back task, subjects were asked to determine whether a played melody (2000ms) was identical to a melody item played after a rest period (2000ms). In the 0-back task, subjects were required to simply signal when the melody had finished playing. Between-groups contrasts (M>NM) were presented for the 1-back task compared to rest. |

**Supplementary table 5.**

*Characteristics of MRI acquisition for studies included in meta-analysis.*

| Author (Year) | Field strength | MRI system | MRI Model | Head coil | T1 Sequence | Repetition time (TR) | Echo time (TE) | Voxel size (mm) | T2 Sequence | Repetition time (TR) | Echo time (TE) | Voxel size (mm) | Analysis Software | Stereotactic Space |
| --- | --- | --- | --- | --- | --- | --- | --- | --- | --- | --- | --- | --- | --- | --- |
| Hoppe (2014) | 1.5T | Siemens | Avanto | 8-channel | MPRAGE | - | - | - | EPI | 2500 ms | 45 ms | 3mm | SPM | MNI |
| Huang (2010) | 1.5T | Siemens | Sonata | Custom | FLASH | 30ms | 1.17ms | - | EPI | 2000 ms | 60 ms |  | AFNI | Talairach |
| Pallesen (2010) | 1.5T | Siemens | Sonata | Birdcage | MPRAGE | 1900ms | 3.86ms | 1x1x1 | EPI | 3660 ms | 40 ms | 3.5x3.5x4 | FSL | MNI |
| Pau (2013) | 3T | Siemens | Magnetom | 12-Channel | MPRAGE | - | - | 1x1x1 | EPI | 2000 ms | 30 ms | 3x3x3 | SPM | MNI |
| Schmithorst (2004) | 3T | Bruker | Medspec | - | T1w | - | - | - | EPI | 3000 ms | 38 ms | 5mm | IDL | Talairach |
| Schulze (2010) | 3T | Siemens | Trio | - | MPRAGE | - | - | - | EPI | 6600 ms | 30 ms | 3x3x4 | LIPSIA | Talairach |
| Schulze (2011) | 3T | Siemens | Trio | - | - | - | - | - | EPI | 6600 ms | 30 ms | 3x3x4 | LIPSIA | Talairach |
| Sluming (2007) | 1.5T | GE | - | - | - | - | - | - | EPI | 3000 ms | 40 ms | 5mm | SPM | MNI |
| Yamashita (2022) | 3T | Siemens | Magnetom | 12-Channel | MPRAGE | - | - | 1x1x1 | EPI | 2000 ms | 25 ms | 3.5x3.5x3.5 | SPM | MNI |

**Supplementary table 6.**

*Meta-analytic connectivity modelling (MACM) results for M>NM and NM>M contrasts at cluster-level inference p < .05.*

| **Musicians > Non-Musicians** | | | | | | | | | | |  | |
| --- | --- | --- | --- | --- | --- | --- | --- | --- | --- | --- | --- | --- |
|  |  |  | MNI Coordinates | | |  |  |  |  |  | |  |
| Seed Region | Cluster number | Volume (mm^3) | x | y | z | ALE | P | Z | Label | Brodmann Area | |  |
| **Right** | 1 | 11760 | 6 | -6 | 60 | 0.1212 | 0 | 14.64 | R.Medial Frontal Gyrus | BA 6 | |  |
| **MedFG** |  |  | 6 | 12 | 40 | 0.0298 | 1.42E-07 | 5.13 | R. Cingulate Gyrus | BA 32 | |  |
| **(M>NM #1)** | 2 | 4368 | 36 | -14 | 52 | 0.0308 | 6.84E-08 | 5.27 | R. Precentral Gyrus | BA 4 | |  |
|  |  |  | 42 | -4 | 56 | 0.0272 | 8.19E-07 | 4.79 | R. Precentral Gyrus | BA 6 | |  |
|  |  |  | 38 | -22 | 54 | 0.0271 | 8.94E-07 | 4.78 | R. Precentral Gyrus | BA 4 | |  |
|  |  |  | 28 | -8 | 60 | 0.0265 | 1.35E-06 | 4.69 | R. Middle Frontal Gyrus | BA 6 | |  |
|  |  |  | 44 | -30 | 46 | 0.0251 | 3.48E-06 | 4.49 | R. Inferior Parietal Lobule | BA 40 | |  |
|  | 3 | 3896 | 14 | -18 | 4 | 0.0308 | 6.89E-08 | 5.27 | R. Thalamus (Medial Dorsal Nucleus) | - | |  |
|  |  |  | 24 | 6 | 2 | 0.0293 | 2.03E-07 | 5.07 | R. Putamen | - | |  |
|  |  |  | 26 | 2 | -8 | 0.0262 | 1.63E-06 | 4.65 | R. Putamen | - | |  |
|  |  |  | 24 | -10 | 0 | 0.0222 | 2.19E-05 | 4.09 | R. Lateral Globus Pallidus | - | |  |
|  | 4 | 2544 | -22 | -10 | 2 | 0.0271 | 9.00E-07 | 4.77 | L. Lateral Globus Pallidus | - | |  |
|  |  |  | -24 | 0 | 2 | 0.0228 | 1.50E-05 | 4.17 | L. Putamen | - | |  |
|  | 5 | 2480 | -56 | 2 | 20 | 0.0349 | 3.42E-09 | 5.79 | L. Precentral Gyrus | BA 6 | |  |
|  |  |  | -52 | 4 | 36 | 0.0236 | 9.14E-06 | 4.28 | L. Precentral Gyrus | BA 6 | |  |
|  | 6 | 1400 | -38 | -8 | 56 | 0.0296 | 1.66E-07 | 5.10 | L. Precentral Gyrus | BA 4 | |  |
|  | 7 | 1096 | 46 | -2 | 10 | 0.0256 | 2.47E-06 | 4.57 | R. Insula | BA 13 | |  |
|  | 8 | 992 | -46 | -24 | 38 | 0.0220 | 2.47E-05 | 4.06 | L. Postcentral Gyrus | BA 2 | |  |
|  |  |  | -50 | -24 | 36 | 0.0213 | 3.94E-05 | 3.95 | L. Postcentral Gyrus | BA 2 | |  |
| Non-musicians > Musicians | | | | | | | | | | |  | |
|  |  |  | MNI Coordinates | | |  |  |  |  |  | |  |
| Seed Region | Cluster number | Volume (mm^3) | x | y | z | ALE | P | Z | Label | Brodmann Area | |  |
| **Right MOG** | 1 | 12192 | -30 | -88 | 2 | 0.0448 | 1.29E-13 | 7.31 | L. Middle Occipital Gyrus | BA 18 | |  |
| **(NM>M #1)** |  |  | -46 | -72 | -8 | 0.0269 | 2.01E-07 | 5.07 | L. Fusiform Gyrus | BA 19 | |  |
|  |  |  | -42 | -70 | -14 | 0.0256 | 5.15E-07 | 4.89 | L. Fusiform Gyrus | BA 19 | |  |
|  |  |  | -48 | -70 | -2 | 0.0253 | 6.57E-07 | 4.84 | L. Inferior Temporal Gyrus | BA 37 | |  |
|  |  |  | -22 | -92 | -12 | 0.0237 | 1.99E-06 | 4.61 | L. Fusiform Gyrus | BA 18 | |  |
|  |  |  | -28 | -88 | 24 | 0.0227 | 3.99E-06 | 4.47 | L. Middle Occipital Gyrus | BA 19 | |  |
|  |  |  | -40 | -84 | -6 | 0.0184 | 6.92E-05 | 3.81 | L. Middle Occipital Gyrus | BA 18 | |  |
|  |  |  | -32 | -84 | -16 | 0.0167 | 2.09E-04 | 3.53 | L. Declive (Cerebellum) | - | |  |
|  | 2 | 4912 | 34 | -90 | 0 | 0.1287 | 0 | 15.67 | R. Middle Occipital Gyrus | BA 18 | |  |
|  | 3 | 4696 | -26 | -52 | 38 | 0.0278 | 1.06E-07 | 5.19 | No Gray Matter found | - | |  |
|  |  |  | -30 | -52 | 50 | 0.0263 | 3.14E-07 | 4.98 | L. Superior Parietal Lobule | BA 7 | |  |
|  |  |  | -24 | -70 | 48 | 0.0238 | 1.87E-06 | 4.63 | L. Precuneus | BA 7 | |  |
|  |  |  | -26 | -70 | 36 | 0.0191 | 4.60E-05 | 3.91 | L. Precuneus | BA 19 | |  |
|  | 4 | 4424 | 44 | -54 | -22 | 0.0260 | 4.09E-07 | 4.93 | R. Culmen (Cerebellum) | - | |  |
|  |  |  | 52 | -68 | -12 | 0.0212 | 1.11E-05 | 4.24 | R. Fusiform Gyrus | BA 37 | |  |
|  |  |  | 48 | -76 | -6 | 0.0200 | 2.52E-05 | 4.05 | R. Inferior Occipital Gyrus | BA 19 | |  |
|  |  |  | 50 | -64 | 2 | 0.0194 | 3.80E-05 | 3.96 | R. Middle Temporal Gyrus | BA 37 | |  |
|  |  |  | 52 | -66 | -2 | 0.0189 | 5.00E-05 | 3.89 | R. Middle Temporal Gyrus | BA 37 | |  |
|  |  |  | 42 | -68 | -6 | 0.0158 | 3.71E-04 | 3.37 | R. Fusiform Gyrus | BA 19 | |  |
|  | 5 | 2136 | -4 | 16 | 46 | 0.0254 | 5.95E-07 | 4.86 | L. Medial Frontal Gyrus | BA 32 | |  |
|  |  |  | 0 | 6 | 58 | 0.0185 | 6.70E-05 | 3.82 | L. Medial Frontal Gyrus | BA 6 | |  |
|  |  |  | 2 | 2 | 60 | 0.0183 | 7.42E-05 | 3.79 | L. Medial Frontal Gyrus | BA 6 | |  |
|  | 6 | 2040 | -46 | 10 | 22 | 0.0233 | 2.66E-06 | 4.55 | L. Inferior Frontal Gyrus | BA 9 | |  |
|  |  |  | -52 | 26 | 32 | 0.0222 | 5.66E-06 | 4.39 | L. Middle Frontal Gyrus | BA 9 | |  |
|  |  |  | -42 | 6 | 30 | 0.0184 | 6.87E-05 | 3.81 | L. Precentral Gyrus | BA 6 | |  |
|  |  |  | -46 | 22 | 26 | 0.0177 | 1.07E-04 | 3.70 | L. Middle Frontal Gyrus | BA 9 | |  |
|  |  |  | -50 | 16 | 32 | 0.0177 | 1.11E-04 | 3.69 | L. Middle Frontal Gyrus | BA 9 | |  |
| **Right PCG** | 1 | 6416 | 38 | -6 | 54 | 0.1188 | 0 | 14.78 | R. Precentral Gyrus | BA 6 | |  |
| **(NM>M #2)** | 2 | 5816 | -4 | -4 | 56 | 0.0367 | 6.65E-11 | 6.42 | L. Medial Frontal Gyrus | BA 6 | |  |
|  |  |  | 0 | 4 | 52 | 0.0299 | 1.44E-08 | 5.55 | L. Medial Frontal Gyrus | BA 6 | |  |
|  | 3 | 2832 | -40 | -12 | 52 | 0.0238 | 1.23E-06 | 4.71 | L. Precentral Gyrus | BA 4 | |  |
|  |  |  | -28 | -8 | 56 | 0.0231 | 2.06E-06 | 4.60 | L. Precentral Gyrus | BA 6 | |  |
|  | 4 | 2608 | -28 | -54 | 56 | 0.0258 | 2.88E-07 | 5.00 | L. Precuneus | BA 7 | |  |
|  |  |  | -32 | -50 | 48 | 0.0241 | 1.01E-06 | 4.75 | L. Inferior Parietal Lobule | BA 40 | |  |
|  |  |  | -38 | -40 | 44 | 0.0192 | 2.95E-05 | 4.02 | L. Inferior Parietal Lobule | BA 40 | |  |
|  | 5 | 1312 | -56 | 4 | 28 | 0.0237 | 1.29E-06 | 4.70 | L. Precentral Gyrus | BA 6 | |  |
|  |  |  | -56 | 4 | 24 | 0.0235 | 1.56E-06 | 4.66 | L. Precentral Gyrus | BA 6 | |  |
|  | 6 | 1224 | -26 | -4 | 0 | 0.0244 | 8.03E-07 | 4.80 | L. Putamen | - | |  |
|  |  |  | -28 | -6 | 10 | 0.0216 | 5.68E-06 | 4.39 | L. Putamen | - | |  |
|  | 7 | 1040 | -14 | -20 | 6 | 0.0301 | 1.21E-08 | 5.58 | L. Thalamus (Ventral Posterior Medial Nucleus) | - | |  |
| **Left IFP** | 1 | 14144 | -54 | -28 | 40 | 0.1328 | 0 | 16.04 | L. Postcentral Gyrus | BA 2 | |  |
| **NM>M #3** |  |  | -44 | -40 | 54 | 0.0238 | 1.76E-06 | 4.64 | L. Inferior Parietal Lobule | BA 40 | |  |
|  |  |  | -28 | -60 | 50 | 0.0234 | 2.34E-06 | 4.58 | L. Superior Parietal Lobule | BA 7 | |  |
|  |  |  | -38 | -44 | 42 | 0.0223 | 4.89E-06 | 4.42 | L. Supramarginal Gyrus | BA 40 | |  |
|  |  |  | -26 | -48 | 42 | 0.0211 | 1.09E-05 | 4.25 | No Gray Matter found | - | |  |
|  |  |  | -40 | -12 | 52 | 0.0177 | 1.06E-04 | 3.70 | L. Precentral Gyrus | BA 4 | |  |
|  | 2 | 5688 | -6 | 10 | 42 | 0.0330 | 1.59E-09 | 5.92 | L. Cingulate Gyrus | BA 24 | |  |
|  |  |  | -2 | 4 | 52 | 0.0313 | 6.16E-09 | 5.70 | L. Medial Frontal Gyrus | BA 6 | |  |
|  |  |  | 8 | 18 | 42 | 0.0172 | 1.53E-04 | 3.61 | R. Cingulate Gyrus | BA 32 | |  |
|  | 3 | 4480 | -52 | 6 | 22 | 0.0357 | 1.87E-10 | 6.26 | L. Inferior Frontal Gyrus | BA 9 | |  |
|  |  |  | -46 | 8 | 10 | 0.0193 | 3.71E-05 | 3.96 | L. Precentral Gyrus | BA 44 | |  |
|  | 4 | 4360 | 56 | -22 | 38 | 0.0307 | 1.00E-08 | 5.61 | R. Postcentral Gyrus | BA 2 | |  |
|  |  |  | 50 | -36 | 46 | 0.0228 | 3.35E-06 | 4.50 | R. Inferior Parietal Lobule | BA 40 | |  |
|  | 5 | 2416 | -14 | -16 | 0 | 0.0268 | 1.90E-07 | 5.08 | L. Thalamus | - | |  |
|  |  |  | 4 | -14 | 10 | 0.0174 | 1.35E-04 | 3.64 | R. Thalamus (Medial Dorsal Nucleus) | - | |  |
|  |  |  | -4 | -16 | 10 | 0.0163 | 2.60E-04 | 3.47 | L. Thalamus (Medial Dorsal Nucleus) | - | |  |
|  |  |  | -8 | -10 | 10 | 0.0158 | 3.65E-04 | 3.38 | L. Thalamus | - | |  |
|  | 6 | 2080 | 30 | -60 | 46 | 0.0248 | 8.11E-07 | 4.80 | R. Superior Parietal Lobule | BA 7 | |  |
|  |  |  | 26 | -64 | 58 | 0.0240 | 1.47E-06 | 4.67 | R. Precuneus | BA 7 | |  |
|  |  |  | 32 | -50 | 52 | 0.0183 | 7.11E-05 | 3.80 | R. Superior Parietal Lobule | BA 7 | |  |
|  | 7 | 1512 | -36 | 16 | 4 | 0.0273 | 1.36E-07 | 5.14 | L. Insula | BA 13 | |  |
|  | 8 | 1456 | -26 | -6 | 56 | 0.0226 | 3.94E-06 | 4.47 | L. Middle Frontal Gyrus | BA 6 | |  |
|  |  |  | -32 | -2 | 46 | 0.0200 | 2.35E-05 | 4.07 | L. Middle Frontal Gyrus | BA 6 | |  |
|  | 9 | 992 | 34 | 18 | 4 | 0.0215 | 8.46E-06 | 4.30 | R. Claustrum | - | |  |
|  |  |  | 42 | 20 | -8 | 0.0155 | 4.43E-04 | 3.32 | R. Insula | BA 13 | |  |
| **Right** | 1 | 8032 | 36 | -10 | 12 | 0.0923 | 4.74E-37 | 12.67 | R. Claustrum | - | |  |
| **Claustrum** |  |  | 56 | -20 | 18 | 0.0172 | 5.07E-05 | 3.89 | R. Insula | BA 40 | |  |
| **NM>M #4** |  |  | 52 | -30 | 24 | 0.0156 | 1.44E-04 | 3.63 | R. Insula | BA 13 | |  |
|  | 2 | 4728 | -36 | -12 | 6 | 0.0243 | 3.19E-07 | 4.98 | L. Claustrum | - | |  |
|  |  |  | -32 | 4 | 6 | 0.0237 | 4.78E-07 | 4.90 | L. Claustrum | - | |  |
|  |  |  | -40 | -16 | 16 | 0.0233 | 6.68E-07 | 4.83 | L. Insula | BA 13 | |  |
|  |  |  | -34 | -2 | 6 | 0.0224 | 1.23E-06 | 4.71 | L. Claustrum | - | |  |
|  |  |  | -38 | 10 | -4 | 0.0153 | 1.80E-04 | 3.57 | L. Claustrum | - | |  |
|  |  |  | -42 | -16 | -2 | 0.0144 | 3.24E-04 | 3.41 | L. Insula | BA 13 | |  |
|  |  |  | -32 | 14 | 4 | 0.0143 | 3.49E-04 | 3.39 | L. Claustrum | - | |  |
|  | 3 | 1456 | -60 | -10 | 12 | 0.0175 | 4.13E-05 | 3.94 | L. Transverse Temporal Gyrus | BA 42 | |  |
|  |  |  | -60 | -4 | 22 | 0.0159 | 1.21E-04 | 3.67 | L. Precentral Gyrus | BA 4 | |  |
|  |  |  | -50 | -6 | 2 | 0.0158 | 1.27E-04 | 3.66 | L. Superior Temporal Gyrus | BA 22 | |  |
|  |  |  | -62 | -4 | 18 | 0.0158 | 1.30E-04 | 3.65 | L. Postcentral Gyrus | BA 43 | |  |
|  | 4 | 1168 | 6 | 16 | 30 | 0.0175 | 4.31E-05 | 3.93 | R. Cingulate Gyrus | BA 24 | |  |
|  |  |  | -6 | 12 | 40 | 0.0171 | 5.45E-05 | 3.87 | L. Cingulate Gyrus | BA 32 | |  |
|  |  |  | -2 | 18 | 32 | 0.0156 | 1.50E-04 | 3.62 | L. Cingulate Gyrus | BA 24 | |  |
|  |  |  | 8 | 24 | 28 | 0.0138 | 4.72E-04 | 3.31 | R. Cingulate Gyrus | BA 32 | |  |
|  | 5 | 1160 | 46 | 8 | 2 | 0.0191 | 1.35E-05 | 4.20 | R. Precentral Gyrus | BA 44 | |  |
|  |  |  | 46 | 12 | -4 | 0.0176 | 3.81E-05 | 3.96 | R. Insula | BA 13 | |  |
|  |  |  | 40 | 8 | -12 | 0.0168 | 6.54E-05 | 3.82 | R. Claustrum | - | |  |
|  | 6 | 1064 | 56 | -6 | 32 | 0.0193 | 1.21E-05 | 4.22 | R. Precentral Gyrus | BA 6 | |  |
|  |  |  | 60 | -6 | 24 | 0.0142 | 3.75E-04 | 3.37 | R. Precentral Gyrus | BA 4 | |  |
|  | 7 | 984 | 12 | -16 | 4 | 0.0259 | 9.10E-08 | 5.22 | R. Thalamus (Medial Dorsal Nucleus) | - | |  |
|  | 8 | 792 | -10 | -18 | 0 | 0.0266 | 5.51E-08 | 5.31 | L. Thalamus (Mammillary Body) | - | |  |
| **Left** | 1 | 13400 | -36 | -70 | -18 | 0.1672 | 0 | 17.32 | L. Declive (Cerebellum) | - | |  |
| **Declive** |  |  | -28 | -90 | 10 | 0.0295 | 5.64E-07 | 4.87 | L. Middle Occipital Gyrus | BA 18 | |  |
| **NM>M #5** |  |  | -24 | -84 | -12 | 0.0279 | 1.56E-06 | 4.66 | L. Fusiform Gyrus | BA 19 | |  |
|  |  |  | -30 | -86 | 16 | 0.0257 | 5.88E-06 | 4.38 | L. Middle Occipital Gyrus | BA 19 | |  |
|  |  |  | -28 | -88 | 2 | 0.0256 | 6.10E-06 | 4.37 | L. Middle Occipital Gyrus | BA 18 | |  |
|  |  |  | -46 | -70 | -4 | 0.0240 | 1.67E-05 | 4.15 | L. Middle Occipital Gyrus | BA 37 | |  |
|  | 2 | 10544 | 42 | -70 | -16 | 0.0439 | 2.56E-11 | 6.57 | R. Declive (Cerebellum) | - | |  |
|  |  |  | 52 | -68 | -2 | 0.0381 | 1.65E-09 | 5.92 | R. Inferior Temporal Gyrus | BA 37 | |  |
|  |  |  | 34 | -62 | -18 | 0.0368 | 4.18E-09 | 5.76 | R. Declive (Cerebellum) | - | |  |
|  |  |  | 36 | -48 | -24 | 0.0337 | 3.44E-08 | 5.39 | R. Culmen (Cerebellum) | - | |  |
|  |  |  | 48 | -60 | -8 | 0.0306 | 2.77E-07 | 5.01 | R. Fusiform Gyrus | BA 37 | |  |
|  |  |  | 26 | -80 | -10 | 0.0205 | 1.25E-04 | 3.66 | R. Occipital Lobe | BA 19 | |  |
|  |  |  | 28 | -74 | -14 | 0.0199 | 1.84E-04 | 3.56 | R. Declive (Cerebellum) | - | |  |
|  | 3 | 6192 | -2 | 24 | 40 | 0.0321 | 1.03E-07 | 5.19 | L. Cingulate Gyrus | BA 32 | |  |
|  |  |  | -2 | 10 | 58 | 0.0319 | 1.15E-07 | 5.17 | L. Medial Frontal Gyrus | BA 6 | |  |
|  |  |  | 8 | 20 | 60 | 0.0271 | 2.49E-06 | 4.57 | R. Superior Frontal Gyrus | BA 6 | |  |
|  |  |  | -4 | 12 | 44 | 0.0186 | 3.63E-04 | 3.38 | L. Cingulate Gyrus | BA 24 | |  |
|  | 4 | 4432 | -44 | 12 | 26 | 0.0367 | 4.39E-09 | 5.75 | L. Inferior Frontal Gyrus | BA 9 | |  |
|  |  |  | -54 | 0 | 34 | 0.0260 | 4.89E-06 | 4.42 | L. Precentral Gyrus | BA 6 | |  |
|  |  |  | -46 | 30 | 16 | 0.0253 | 7.60E-06 | 4.33 | L. Middle Frontal Gyrus | BA 46 | |  |
|  |  |  | -44 | 4 | 42 | 0.0233 | 2.48E-05 | 4.06 | L. Middle Frontal Gyrus | BA 6 | |  |
|  | 5 | 1584 | 44 | -44 | 48 | 0.0293 | 6.37E-07 | 4.84 | R. Inferior Parietal Lobule | BA 40 | |  |
|  |  |  | 44 | -32 | 40 | 0.0275 | 1.99E-06 | 4.61 | R. Inferior Parietal Lobule | BA 40 | |  |
|  | 6 | 1424 | -32 | 22 | 0 | 0.0373 | 3.04E-09 | 5.81 | L. Claustrum | - | |  |
|  | 7 | 1376 | 52 | 14 | 22 | 0.0307 | 2.47E-07 | 5.03 | R. Inferior Frontal Gyrus | BA 9 | |  |
|  | 8 | 1208 | 34 | 22 | -4 | 0.0316 | 1.37E-07 | 5.14 | R. Claustrum | - | |  |
|  | 9 | 1184 | 32 | -90 | 4 | 0.0250 | 9.06E-06 | 4.29 | R. Middle Occipital Gyrus | - | |  |
|  |  |  | 38 | -82 | 4 | 0.0218 | 6.07E-05 | 3.84 | R. Inferior Occipital Gyrus | BA 19 | |  |
|  | 10 | 1080 | -28 | -58 | 52 | 0.0237 | 1.94E-05 | 4.11 | L. Superior Parietal Lobule | BA 7 | |  |
|  |  |  | -24 | -70 | 52 | 0.0229 | 3.21E-05 | 4.00 | L. Superior Parietal Lobule | BA 7 | |  |
|  |  |  | -28 | -54 | 42 | 0.0205 | 1.27E-04 | 3.66 | L. Angular Gyrus | BA 39 | |  |

*Note: MedFG = Medial Frontal Gyrus, MOG = Middle Occipital Gyrus, PCG = Precentral Gyrus, IPL = Inferior Parietal Lobule*
